# A TERRA-NONO axis drives fibroblast reprogramming in cancer

**DOI:** 10.1126/sciadv.aed3358

**Published:** 2026-10-02

**Authors:** Emery Di Cicco, Jovan Isma, Stefano Sol, Gaetana Restivo, Luigi Mazzeo, Atul Katarkar, Sandro Goruppi, Joni Renee White, Juerg Hafner, Victor A. Neel, Anna Mandinova, Mitchell P. Levesque, Gian Paolo Dotto

**Affiliations:** ^1^ Cutaneous Biology Research Center, Department of Dermatology, Massachusetts General Hospital and Harvard Medical School, Charlestown, MA 02129, USA.; ^2^ Department of Immunobiology, University of Lausanne, Epalinges, Switzerland.; ^3^ Department of Dermatology, University Hospital Zurich, University of Zurich, Zurich, Switzerland.; ^4^ Department of Dermatology, Massachusetts General Hospital and Harvard Medical School, Boston, MA 02114, USA.; ^5^ International Cancer Prevention Institute, Epalinges, Switzerland.

## Abstract

The molecular mechanisms linking chromosome homeostasis to fibroblast reprogramming into cancer-associated fibroblasts (CAFs) remain poorly understood. Here, we identify a clinically relevant, telomere-associated nuclear pathway driving CAF activation across multiple human cancers. The long noncoding RNA TERRA is consistently elevated in CAFs from skin, lung, and breast tumors. We show that the androgen receptor (AR) represses TERRA transcription through subtelomeric binding, and its loss enables TERRA up-regulation and interaction with the RNA binding protein NONO. This interaction forms a CAF-specific nuclear complex that reprograms gene expression. Mechanistically, NONO is directly recruited to and binds the promoters of key CAF effector genes, where it regulates their transcription in a TERRA-dependent manner. Disrupting the TERRA-NONO axis, via TERRA silencing or pharmacologic NONO inhibition, reverses CAF activation and suppresses tumor-stroma interactions in vitro and in vivo, highlighting its therapeutic potential.

## INTRODUCTION

The rising incidence of cancer with age is driven by converging biological processes, including telomere alterations at chromosome ends and epigenetic reprogramming within both cancer cells and their microenvironment (*1*). Whether these hallmarks are functionally interconnected remains an open question.

Accumulating evidence indicates that stromal fibroblasts also undergo telomere dysfunction during tumorigenesis. In particular, our previous studies demonstrated that the transition of human dermal fibroblasts (HDFs) into cancer-associated fibroblasts (CAFs) is associated with alterations in telomere-protective complexes, increased telomeric DNA damage, and chromosome-end abnormalities, occurring in the absence of overt changes in telomere length (*2*, *3*).

The long noncoding telomeric repeat-containing RNA (RNA TERRA) is a critical regulator of telomere function. It modulates telomeric chromatin architecture, influences telomerase activity, and participates in the alternative lengthening of telomere pathway (*4*). Beyond telomeres, TERRA associates with internal chromosomal loci and interacts with chromatin remodelers and transcriptional regulators, suggesting its role in broader epigenetic control (*5*, *6*).

Among TERRA’s binding partners is NONO (also known as p54nrb), a multifunctional member of the *Drosophila* behavior/human splicing protein family. NONO functions as a molecular scaffold in processes ranging from transcription and RNA processing to genome maintenance (*7*). At telomeres, NONO binds TERRA and preserves their integrity by suppressing the formation of harmful RNA:DNA R-loops (*8*). However, the role of the TERRA-NONO complex beyond telomeres remains poorly defined. TERRA transcripts range from 100 base pairs to more than 9 kb and originate from subtelomeric regions across multiple chromosomes (*4*). TERRA has been implicated in telomere length control in either a positive or negative fashion in connection with telomerase activity (*9*, *10*). Its expression varies notably across various cancer cell types, being both up-regulated and down-regulated, suggesting context-dependent functions (*11*). Whether or not TERRA plays an intrinsic role in stromal fibroblasts and their activation into CAFs, key stromal players that drive tumor progression (*12*–*14*), has not been explored.

Environmental damaging agents such as UVA radiation and cigarette smoke can penetrate the stromal compartment and induce long-lasting protumorigenic changes. In human skin, this has been shown to result in down-regulation of the androgen receptor (AR), which promotes senescence and conversion of HDFs into CAFs (*15*, *16*).

Here, we demonstrate that TERRA, a long noncoding RNA transcribed from multiple subtelomeric regions, is under direct negative regulation by AR in HDFs and is consistently up-regulated in CAFs across major skin cancer types, as well as in lung and breast cancer. We find that TERRA functions as both a necessary and sufficient driver of CAF transcriptional reprogramming. Quantitative proteomic analysis identifies the RNA binding protein NONO as a key effector of TERRA function, which is recruited by TERRA to the promoters of CAF genes, where it directly binds and regulates their transcription. Silencing NONO recapitulates the effects of TERRA depletion in reversing the CAF gene expression program, while pharmacological disruption of the TERRA-NONO complex abrogates CAF activation and suppresses tumorigenesis. Together, these findings uncover a previously unrecognized role for TERRA-NONO complex in governing stromal plasticity and reveal a therapeutically targetable mechanism of CAF reprogramming in the tumor microenvironment.

## RESULTS

### TERRA is up-regulated in CAFs across multiple tumor types

The expression and role of the telomeric long noncoding RNA TERRA in the tumor stroma remain largely undefined. TERRA is transcribed from the subtelomeric regions of most human chromosomes (*4*). Using reverse transcription quantitative polymerase chain reaction (RT-qPCR) analysis with subtelomeric primers (1q, 2q, 7p, 9q, 13q, 17p, and XqYq), we detected consistently elevated TERRA levels in six CAF strains derived from cutaneous squamous cell carcinomas (SCCs) compared with that in matched HDFs from nonaffected adjacent skin, with the highest level of TERRA transcription from chromosome 7p (Fig. 1A and fig. S1, A and B). TERRA was concordantly induced with multiple canonical CAF markers, as previously reported. (*15*, *16*). Increased TERRA levels were also found in CAFs from basal cell carcinomas (BCAFs) and melanomas (MCAFs) when compared with those in reference HDFs from nondiseased areas (Fig. 1B and fig. S1C).

**Fig. 1.**
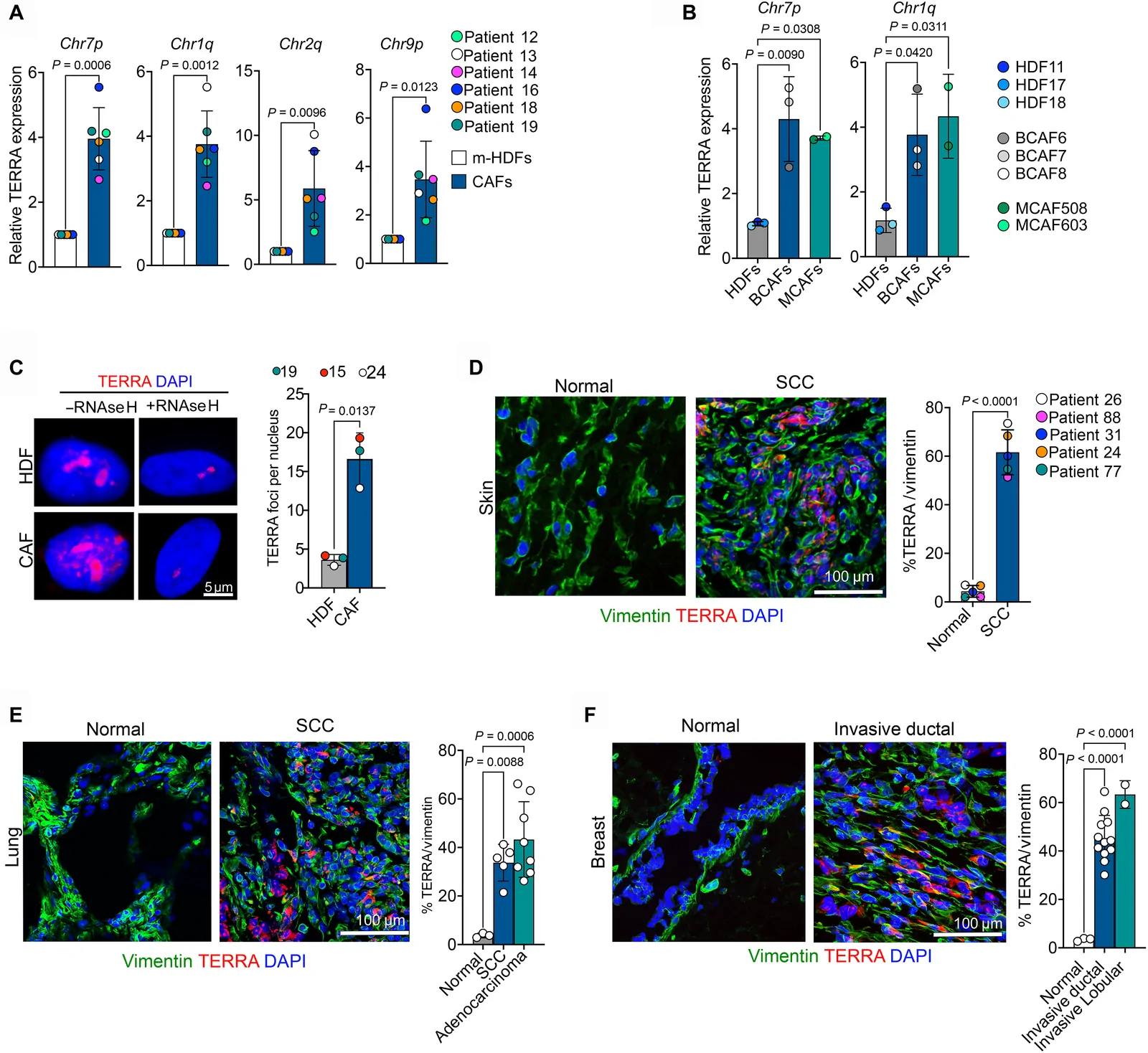
TERRA is highly expressed in CAFs across multiple tumor types. (**A**) Chromosome-specific TERRA expression in CAFs isolated from cutaneous SCC (cSCC) lesions compared with that in matched HDFs from the same patients (#12, #13, #14, #16, #18, and #19), assessed by RT-qPCR using chromosome-specific primers. Expression is shown as fold change in CAFs relative to HDFs after normalization to glyceraldehyde-3-phosphate dehydrogenase (GAPDH; *n* = 6 strains). Data are means ± SD; two-tailed paired *t* test. (**B**) RT-qPCR analysis of TERRA expression in CAFs derived from BCC (BCAF6, BCAF7, and BCAF8; *n* = 3) and melanoma (MCAF508 and MCAF603; *n* = 2) relative to age-matched HDFs (HDF11, HDF17, and HDF18; *n* = 3). Data are means ± SD; one-way analysis of variance (ANOVA) with Dunnett’s multiple comparisons test. (**C**) Representative RNA-FISH images showing TERRA (red) in CAFs and matched HDFs, with 4′,6-diamidino-2-phenylindole (DAPI) nuclear counterstaining (blue). RNase H treatment (5 U) was used to confirm probe specificity. Quantification was performed per nucleus (>40 cells per strain), with values averaged to generate one data point per strain (*n* = 3 strains). Means ± SD; paired *t* test. (**D**) Combined IF/RNA-FISH analysis of SCC patient samples using TERRA probe (red), vimentin (green), and DAPI (blue). Quantification shows the percentage of vimentin-positive cells with TERRA foci across four fields per patient (*n* = 5 patients). Means ± SD; paired *t* test. (**E** and **F**) IF/RNA-FISH analysis of lung and breast tumor tissue microarrays. Quantification represents the percentage of vimentin-positive cells with TERRA foci per section (lung: *n* = 3 normal, *n* = 5 SCC, and *n* = 8 adenocarcinoma; breast: *n* = 3 normal, *n* = 12 IDC, and *n* = 2 ILC). Means ± SD; one-way ANOVA with Dunnett’s test.

We further assessed TERRA expression by RNA–fluorescence in situ hybridization (FISH) using a probe targeting the repetitive TERRA sequence, with ribonuclease H (RNase H) treatment serving as a specificity control (*17*). Compared with matched HDFs, multiple CAF strains exhibited a marked increase in TERRA foci, which fell into two broad size classes, small and large, with some interstrain heterogeneity (Fig. 1C). Notably, the pattern of TERRA signal detected in these primary cells differs from the predominantly small foci previously described in established cancer cell lines such as HeLa or U2OS (*8*, *18*, *19*). As shown later in the manuscript, the smaller foci preferentially colocalized with telomeres, whereas the larger foci were more frequently associated with the nucleolar compartment. To validate these findings in patient specimens, immuno-RNA-FISH performed on five human cSCC biopsies revealed significantly increased TERRA accumulation in vimentin-positive stromal fibroblasts relative to adjacent normal skin (Fig. 1D and fig. S1, D and E). Notably, this pattern was recapitulated in a tissue microarray of 37 primary lung SCC and adenocarcinoma samples, where vimentin-positive stromal cells displayed significantly elevated TERRA foci compared with that in matched normal lung tissue (Fig. 1E and fig. S1F). A parallel analysis of breast cancer tissue microarray showed a notable up-regulation of TERRA in stromal fibroblasts (Fig. 1F and fig. S1G). Collectively, these findings establish TERRA up-regulation in CAFs as a conserved hallmark of the tumor stroma in skin, lung, and breast cancers, underscoring its potential as a broadly applicable biomarker and mechanistic driver of stromal reprogramming.

### TERRA is negatively regulated by the AR

Down-regulation of AR expression provides a well-characterized model for the early steps of CAF activation (*15*, *16*, *20*). Sequence analysis of multiple subtelomeric regions revealed the presence of canonical AR binding sites located in proximity to TERRA transcriptional start sites (Fig. 2A, top). TERRA is transcribed by RNA polymerase II from subtelomeric promoters, with transcription initiating in these regions and extending into telomeric repeats (*21*, *22*). Chromatin immunoprecipitation (ChIP) combined with Tn5 transposase tagging (“ChIPmentation”) (*16*) with anti-AR antibodies with high efficiency of AR pull down (fig. S2A) showed consistent binding of AR to most of these regions in three independent HDF strains (Fig. 2A, bottom).

**Fig. 2.**
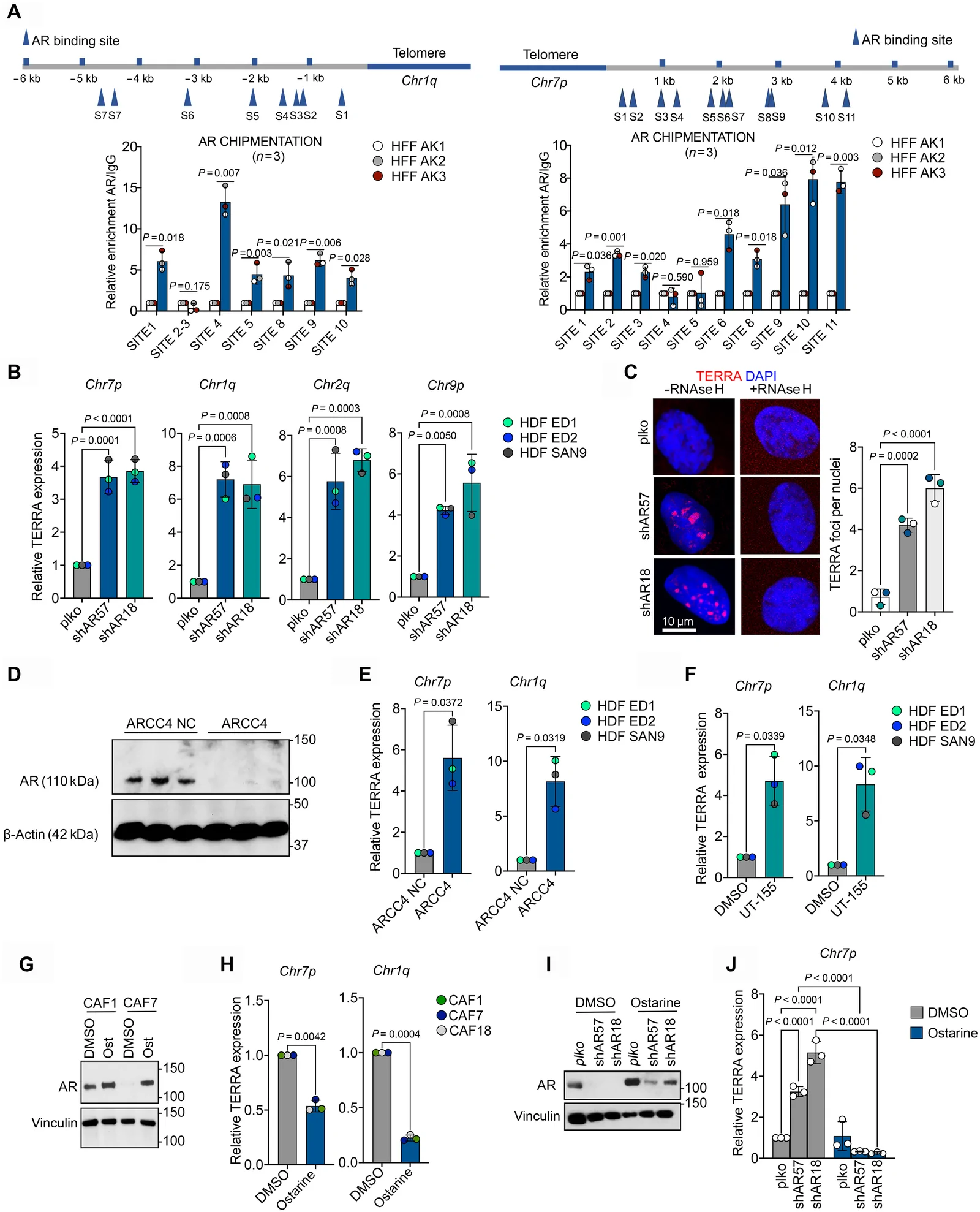
AR is a negative regulator of TERRA transcription. (**A**) Top: Schematic of predicted AR binding sites (blue arrowheads) within ∼6-kb subtelomeric regions of indicated chromosomes. Bottom: ChIPmentation-qPCR analysis showing AR occupancy at these sites in three independent HDF strains (AK1, AK2, and AK3), expressed as fold enrichment over immunoglobulin G (IgG) controls (*n* = 3). Means ± SD; paired *t* test. (**B**) RT-qPCR analysis of chromosome-specific TERRA expression in HDFs following AR silencing using two independent short hairpin RNAs (shRNAs; shAR#57 and shAR#18) compared with control (plko). Data are normalized to GAPDH and expressed as fold change relative to control (*n* = 3 strains). Means ± SD; one-way ANOVA with Dunnett’s test. (**C**) Representative RNA-FISH images and quantification of TERRA nuclear foci per cell in AR-silenced HDFs versus control. RNase H (5 U) was used as specificity control. Quantification was performed per nucleus (>40 cells per replicate; *n* = 3 biological replicates). Means ± SD; one-way ANOVA with Dunnett’s test. (**D**) Immunoblot analysis of AR levels in HDFs treated with AR degrader ARCC4 (1 μM, 48 hours) or inactive control. (**E** and **F**) RT-qPCR analysis showing increased TERRA expression in HDFs treated with ARCC4 or UT-155 (1 μM, 48 hours) relative to controls (*n* = 3 strains). Means ± SD; paired two-tailed *t* test. (**G** and **H**) Immunoblot and RT-qPCR analyses demonstrating reduced TERRA expression in CAFs treated with AR agonist ostarine (Ost; 10 μM, 48 hours) (*n* = 3 strains). Means ± SD; paired *t* test. (**I** and **J**) AR-dependent regulation confirmed by loss of ostarine responsiveness upon AR silencing. Means ± SD; one-way ANOVA with Šidák’s test (*n* = 3).

Functionally, AR silencing in three HDF strains (fig. S3A) resulted in a significant increase in TERRA expression from multiple chromosomes (Fig. 2B and fig. S3B), with RNA-FISH showing increased nuclear TERRA foci, both large and small, that phenocopied the CAF profile (Fig. 2C). TERRA levels inversely correlate with endogenous AR protein abundance across CAF strains (fig. S3G).

To further validate the link between AR activity and TERRA regulation, we used pharmacologic strategies to inhibit AR function. Treatment with ARCC4, a PROTAC compound that induces AR protein degradation (Fig. 2D) (*23*), led to elevated TERRA expression (Fig. 2E and fig. S3C), with similar effects being elicited by treatment with UT-155, a selective AR antagonist that suppresses AR transcriptional activity (*24*), inducing CAF effector genes (Fig. 2F and fig. S3, D to F). Conversely, treatment of CAFs with ostarine, a selective AR agonist (*25*) that can enhance AR levels via a positive feedback mechanism, reducing CAF activation (Fig. 2G) (*20*), resulted in marked suppression of TERRA expression (Fig. 2H and fig. S3G). Suppression of TERRA expression was also observed after ostarine treatment of HDFs with short hairpin RNA (shRNA)–mediated AR gene silencing (Fig. 2I), which resulted also in an up-regulation of endogenous AR expression (Fig. 2J and fig. S3I). These findings establish AR as a negative regulator of TERRA transcription and demonstrate that AR loss, characteristic of CAFs, directly contributes to TERRA up-regulation and fibroblast reprogramming.

### TERRA exerts dual telomeric and transcriptional functions during fibroblast reprogramming

To investigate the functional role of TERRA in the reprogramming of fibroblasts into CAF, we used both loss- and gain-of-function approaches. TERRA was silenced in CAFs using antisense oligonucleotides (TERRA ASOs) (*5*) and overexpressed in HDFs using a lentiviral vector (*26*). TERRA knockdown by TERRA ASO transfection led to a clear reduction in TERRA levels across multiple subtelomeric regions compared with scrambled controls (SCR ASO) (Fig. 3A and fig. S4A) along with a significant decrease in nuclear TERRA foci (Fig. 3B). In contrast, overexpression in HDFs increased TERRA foci to levels comparable with those seen in CAFs (Fig. 3C). Notably, neither TERRA silencing nor overexpression affected cellular proliferation (fig. S4, B and C).

**Fig. 3.**
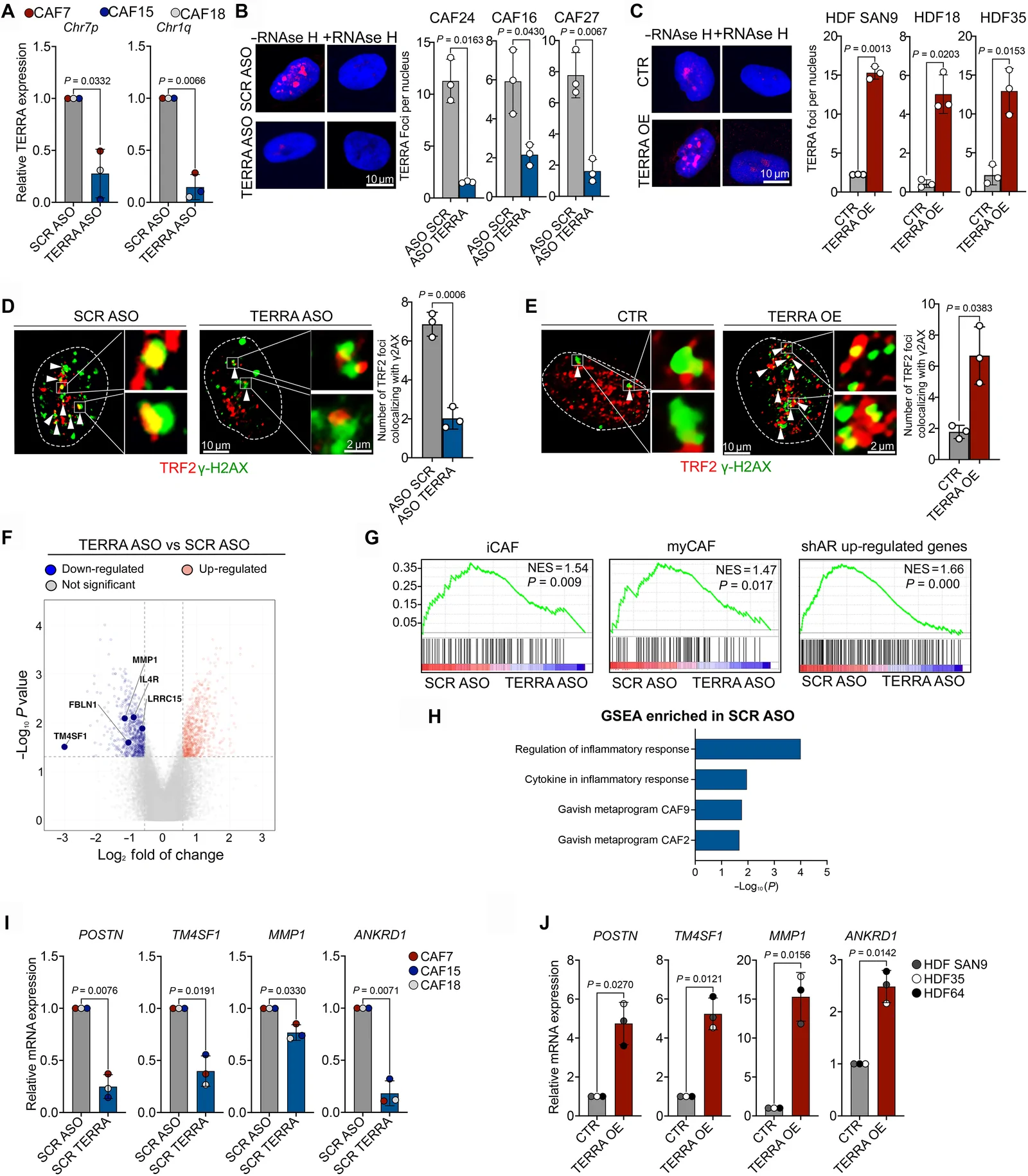
TERRA regulates telomere-associated dysfunction in fibroblasts and CAF transcriptional program. (**A**) Chromosome-specific TERRA expression in CAFs 48 hours after transfection with TERRA ASO or scrambled control (100 nM), assessed by RT-qPCR and normalized to GAPDH (*n* = 3 strains). Means ± SD; paired two-tailed *t* test. (**B**) RNA-FISH analysis of TERRA (red) in CAFs transfected as in (A), with RNase H (5 U) as specificity control. Quantification of nuclear foci was performed per cell (>40 cells per replicate), averaged to one value per replicate (*n* = 3 biological replicates per strain). Means ± SD; paired *t* test. (**C**) RNA-FISH analysis of TERRA foci in HDFs expressing TERRA (TERRA OE) versus control (CTR), with RNase H validation. Quantification was performed per nucleus (>50 cells per replicate; *n* = 3 strains, 3 biological replicates each). Means ± SD; paired *t* test. (**D** and **E**) Combined IF for TRF2 (telomeres) and γH2AX (DNA damage) in CAFs (D) and HDFs (E) following TERRA depletion or overexpression. Insets highlight colocalization. Quantification shows TRF2/γH2AX colocalizing foci per nucleus (>40 cells per replicate; *n* = 3 biological replicates). Means ± SD; paired *t* test. (**F**) Transcriptomic profiling of three CAF strains with TERRA ASO or SCR ASO. Volcano plot displays differential gene expression (*P* < 0.05, |log_2_ fold change| ≥ 0.58) between TERRA ASO and SCR ASO conditions. (**G** and **H**) Gene set enrichment analysis (GSEA) of expression profiles from three CAF strains treated with TERRA ASO versus SCR ASO, analyzed against gene signatures associated with inflammatory CAFs (iCAFs), myofibroblastic CAFs (myCAFs), and genes up-regulated in HDFs upon AR silencing (*16*) and CAF metaprogram CAF9 and CAF2 (*56*), and “Regulation of inflammatory response” (GO:0050727) and “Cytokine production involved in inflammatory response” (GO:0002534). Normalized enrichment scores (NES) and *P* values are shown. (**I** and **J**) RT-qPCR validation of CAF effector genes in CAFs with TERRA silencing and HDFs with TERRA overexpression (*n* = 3 strains). Data normalized to *RPLP0*. Means ± SD; paired two-tailed *t* test.

We next asked whether TERRA contributes to the telomere-associated DNA damage observed in CAFs (*2*, *3*). In line with prior reports linking TERRA dysregulation to telomeric dysfunction (*4*, *5*), double immunofluorescence with antibodies against TRF2 (*6*) and γ-H2AX showed that TERRA silencing in CAFs reduced the telomeric damage response, whereas TERRA overexpression in HDFs had the opposite effect (Fig. 3, D and E). Notably, γ-H2AX foci were modulated in parallel at both telomeric and nontelomeric sites, in line with emerging evidence that TERRA may contribute to a broader DNA damage response through mechanisms that remain to be defined (*27*).

In parallel, we examined the impact of TERRA on transcriptional programs associated with CAF activation. Global transcriptomic analysis revealed a core set of genes consistently up- or down-regulated across three independent CAF strains following TERRA silencing (Fig. 3F). Several established CAF effector genes, including *LRRC15* (*28*), *MMP1* (*29*), *IL4R* (*30*), and *FLN1* (*31*), were significantly down-regulated upon TERRA depletion. *TM4SF1*, which codes for a glycoprotein involved in cancer cell motility and proliferation, was recently identified as a CAF effector involved in both skin (*32*) and gastric (*33*) cancer. It was among the most strongly suppressed (Fig. 3F).

Gene set enrichment analysis (GSEA) further showed that TERRA knockdown coordinately down-regulated multiple CAF-related gene signatures, including transcriptional programs associated with inflammatory and myofibroblastic (*34*) and CAF subtypes identified through a broad analysis of intratumor heterogeneity across multiple cancer type, which are also linked to AR loss (Fig. 3G) (*16*, *20*). Additional functionally relevant gene signatures were likewise affected by TERRA silencing (Fig. 3H and fig. S4C).

Direct RT-qPCR analysis confirmed that TERRA silencing reduced the expression of key CAF effectors, including *POSTN*, *MMP1*, and *TM4SF1*, as well as *ANKRD1*, which encodes a transcriptional coactivator implicated in CAF activation (Fig. 3I) (*16*). Conversely, ectopic TERRA overexpression in HDFs induced up-regulation of these same genes (Fig. 3J). Overexpression of TERRA in CRISPR p53-deficient HDFs, in which the canonical DNA damage response is compromised (*35*, *36*), induced CAF effector genes to a similar extent as in control cells (fig. S5, A and B), indicating that TERRA-driven transcriptional reprogramming occurs independently of p53-mediated DNA damage signaling. Together, these findings support a model in which TERRA exerts a dual role in CAFs, contributing to telomeric dysfunction while driving transcriptional reprogramming programs associated with fibroblast activation.

### TERRA promotes the protumorigenic activity of CAFs

A hallmark of CAFs is their ability to promote the proliferation and tumorigenicity of adjacent cancer cells. To investigate the functional significance of TERRA in this context, we initially used a cancer–stromal cell coculture assay embedded in a thin Matrigel layer (*15*). The expansion of squamous carcinoma cells (SCC13) was markedly reduced in the presence of TERRA-silenced CAFs relative to controls (Fig. 4A), whereas it was enhanced in the presence of TERRA-overexpressing HDFs (Fig. 4B).

**Fig. 4.**
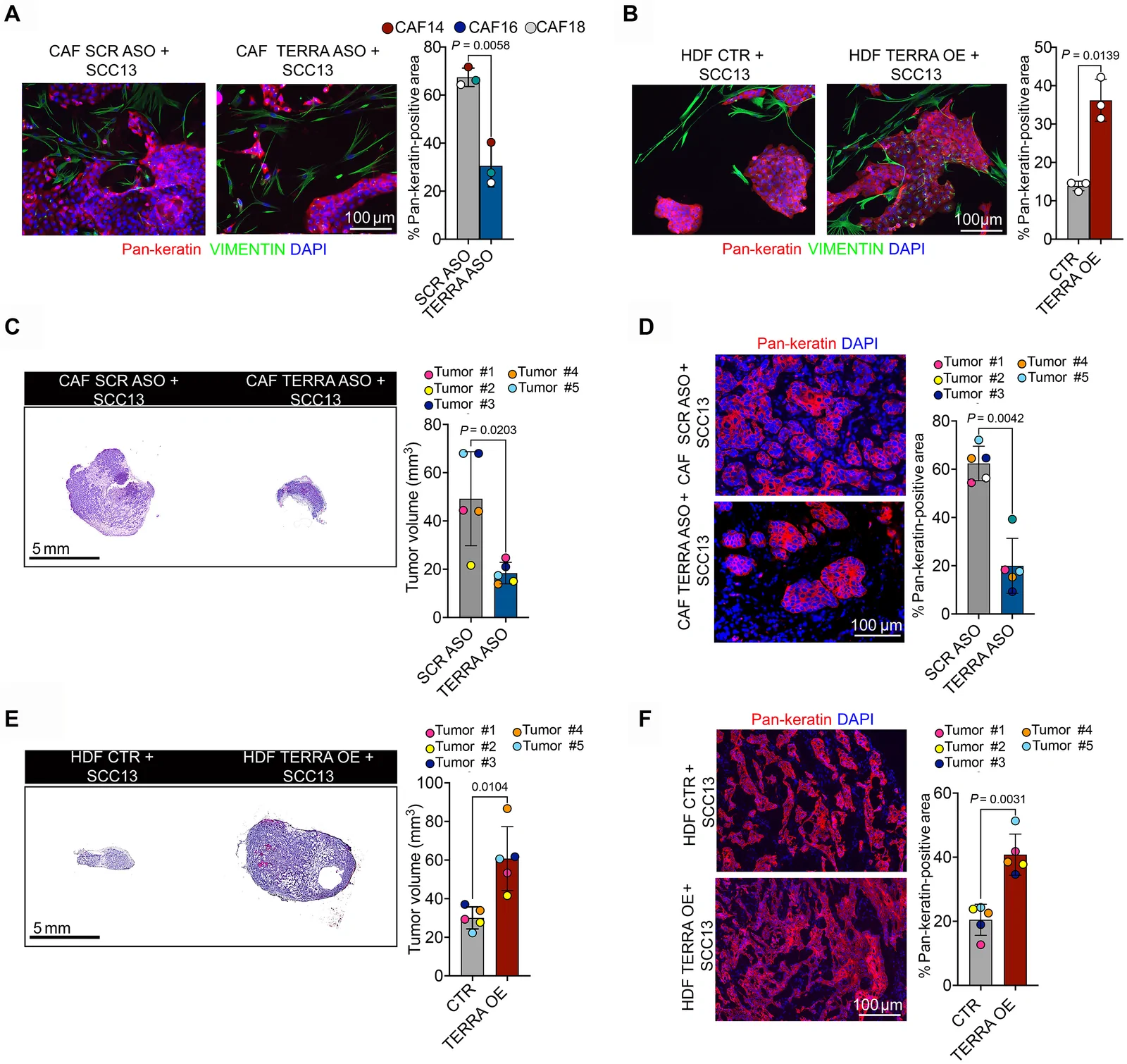
TERRA is required for the tumor-promoting activity of CAFs. (**A**) SCC13 expansion assays in coculture with CAFs transfected with TERRA ASO or scrambled control (100 nM) in a thin Matrigel layer. Representative IF images show SCC cells (PAN-KERATIN, red) and CAFs (vimentin, green). Quantification of tumor cell expansion is expressed as percentage of pan-keratin–positive area (*n* = 3 CAF strains; three wells per strain). Means ± SD; paired *t* test. (**B**) SCC13 expansion assays in coculture with HDFs expressing TERRA (TERRA OE) or control (CTR) under the same conditions. Representative images and quantification are shown (*n* = 3 biological replicates; three wells per replicate). Means ± SD; paired *t* test. (**C**) Intradermal tumorigenicity assays with SCC13 cells coinjected with CAFs transfected with TERRA ASO or control into contralateral mouse back skin. Representative hematoxylin and eosin (H&E) sections and quantification of tumor volume [cubic millimeters; (*L* × *W*^2^)/2] are shown (*n* = 5 tumor pairs). Means ± SD; paired two-tailed *t* test. (**D**) Cancer cell density in tumors from (C), assessed by pan-keratin IF and quantified as percentage of positive area per lesion (*n* = 5 tumor pairs). Means ± SD; paired *t* test. (**E**) Intradermal tumorigenicity assays with SCC13 cells coinjected with HDFs expressing TERRA or control, with representative H&E images and tumor volume quantification (*n* = 5 tumor pairs). Means ± SD; paired two-tailed *t* test. (**F**) Cancer cell density in tumors from (E), quantified by PAN-KERATIN staining (*n* = 5 tumor pairs). Means ± SD; paired *t* test.

In an orthotopic cancer model involving intradermal injection of cells into the back skin of mice, tumors formed by SCC cells coinjected with TERRA-silenced CAFs were significantly smaller and exhibited lower cancer cell density compared with those formed with control CAFs (Fig. 4, C and D). Conversely, SCC cells coinjected with TERRA-overexpressing HDFs gave rise to larger, more cellular tumors than those admixed with control HDFs (Fig. 4, E and F). These findings demonstrate that elevated TERRA expression in CAFs promotes cancer cell proliferation and tumor growth.

### NONO differentially associates with TERRA and AR in CAFs

To identify TERRA-interacting proteins that may contribute to CAF activation, we performed identification of direct RNA-interacting proteins (iDRIP) (*5*) in CAFs using an antisense probe to pull down TERRA and a sense probe as a negative control. Quantitative mass spectrometry across two biological replicates identified 19 proteins significantly enriched in TERRA pulldowns (data file S1), predominantly RNA binding proteins involved in transcription, splicing, and ribosome biology, and others related to cytoskeletal organization (fig. S6A). Among the enriched proteins, we focused on NONO, a multifunctional RNA and DNA binding protein previously shown to interact with TERRA and dampen its impact on telomeres (*8*). STRING network analysis (https://string-db.org/) revealed high-confidence interactions between NONO and several other RNA binding proteins identified in the pulldown (Fig. 5A), consistent with its role as a molecular scaffold involved in transcription, RNA processing, and genome stability (*7*). RNA immunoprecipitation (RIP) using NONO-specific antibodies confirmed its binding to TERRA transcripts across multiple CAF strains (Fig. 5, B and C). For these analyses, we focused on TERRA derived from the chromosome 7p locus as our profiling of multiple subtelomeric regions (fig. S1B) showed that 7p-derived TERRA is expressed at higher levels in CAFs and in HDFs upon AR silencing compared with other loci.

**Fig. 5.**
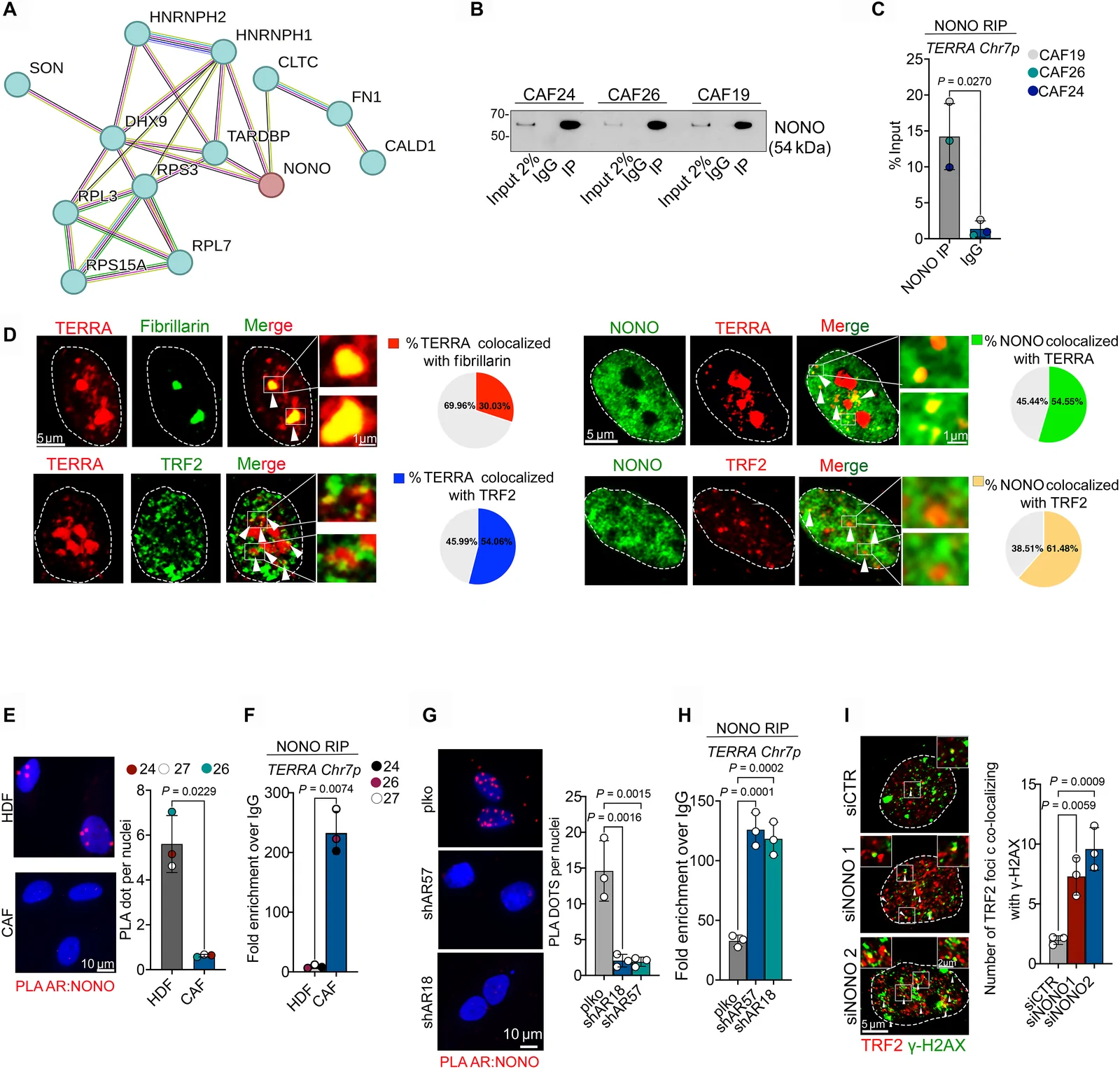
NONO associates with TERRA and AR in CAFs versus HDFs. (**A**) STRING network of TERRA-associated proteins (confidence score ≥ 0.4). NONO is highlighted in red. (**B**) NONO RIP followed by immunoblotting in three CAF strains (CAF19, CAF24, and CAF26). IgG controls and 2% input are shown. (**C**) NONO RIP–RT-qPCR for Chr7p TERRA in CAFs, expressed as percentage of input (*n* = 3 strains). (**D**) TERRA RNA-FISH (red) combined with IF for NONO, TRF2, or fibrillarin (green) in CAFs. Insets show colocalization (yellow); graphs and pie charts quantify NONO colocalization with TERRA or TRF2 and TERRA colocalization with fibrillarin or TRF2. (**E**) PLA of endogenous AR-NONO complexes in CAFs and matched HDFs, quantified as puncta per nucleus (>50 nuclei per strain; *n* = 3). (**F**) NONO RIP–RT-qPCR for TERRA in CAFs and matched HDFs, expressed as fold enrichment over IgG (*n* = 3 strains). (**G**) AR-NONO PLA in HDFs expressing control shRNA or either of two AR-targeting shRNAs (shAR57 and shAR18; >40 cells per replicate; *n* = 3). (**H**) NONO RIP–RT-qPCR for TERRA after AR silencing (*n* = 3). (**I**) TRF2/γH2AX IF colocalization after NONO silencing in CAFs (>40 cells per replicate; *n* = 3). Data are means ± SD. Statistical significance was assessed by paired two-tailed *t* test (C, E, and F) or one-way ANOVA with Dunnett’s test (G to I).

To define the nuclear localization of TERRA and NONO, we performed combined RNA-FISH and immunofluorescence coupled with super-resolution (SoRa) microscopy (Fig. 5D). TERRA RNA-FISH combined with immunofluorescence for the nucleolar marker fibrillarin and the telomeric marker TRF2 indicated that ∼30% of TERRA foci colocalized with nucleoli, whereas a larger fraction (∼54%) colocalized with telomeres (Fig. 5D, right). This distribution was size dependent: Larger TERRA foci preferentially colocalized with nucleoli, whereas smaller, more discrete foci more frequently colocalized with telomeres. TERRA RNA-FISH combined with immunofluorescence for NONO showed that ∼54.5% of NONO signal colocalized with TERRA. Like TERRA, NONO also partially colocalized with the telomeric protein TRF2 (∼61.5%), consistent with its functions at both telomeric and nontelomeric sites (*7*, *8*). TERRA-NONO colocalization was observed predominantly outside nucleoli, within smaller nucleoplasmic foci that frequently overlapped with TRF2-positive sites.

NONO has previously been shown to associate with AR in prostate cancer cells to regulate gene transcription (*37*, *38*). We asked whether a similar interaction occurs in HDFs and is altered during CAF activation. NONO protein levels remained unchanged in CAFs relative to matched HDFs and were similarly unaffected by AR silencing in HDFs (fig. S6, B and C). By contrast, proximity ligation assays (PLA) revealed abundant nuclear NONO-AR complexes in HDFs, which were markedly reduced both in CAFs and in AR-silenced HDFs (Fig. 5, E and G). This loss was accompanied by a reciprocal increase in NONO-TERRA interactions (Fig. 5, F and H), showing that, during CAF activation, NONO shifts from association with AR to association with TERRA. Together, these findings identify NONO as a TERRA-binding partner whose interactions shift during CAF activation from AR toward TERRA, suggesting a role in the CAF reprogramming process.

### NONO is a determinant of the transcriptional program of CAF activation

To investigate the functional role of NONO in CAFs, we performed small interfering RNA (siRNA)–mediated silencing, achieving efficient knockdown within 72 hours (fig. S6D). Previous studies in U2-OS cells have shown that NONO limits the accumulation of TERRA-induced RNA:DNA hybrids (R-loops), thereby protecting against telomere-associated damage (*8*). Consistent with this role, double immunofluorescence analysis with antibodies against TRF2 and γ-H2AX showed that NONO silencing in CAFs, in which TERRA levels are elevated, increased telomere-associated damage (Fig. 5I) (*8*).

We next asked whether NONO also contributes to transcriptional regulation in CAFs. Transcriptomic profiling of multiple CAF strains following NONO depletion using two independent siRNAs revealed widespread transcriptional alterations, including down-regulation of established CAF effector genes (*POSTN*, *INHBA*, and *TM4SF1*) and transcriptional regulators of CAF identity such as *ANKRD1* (Fig. 6A) (*16*), closely resembling the effects of TERRA silencing (Fig. 3F). These changes were validated by RT-qPCR and immunoblotting (fig. S7, A and B).

**Fig. 6.**
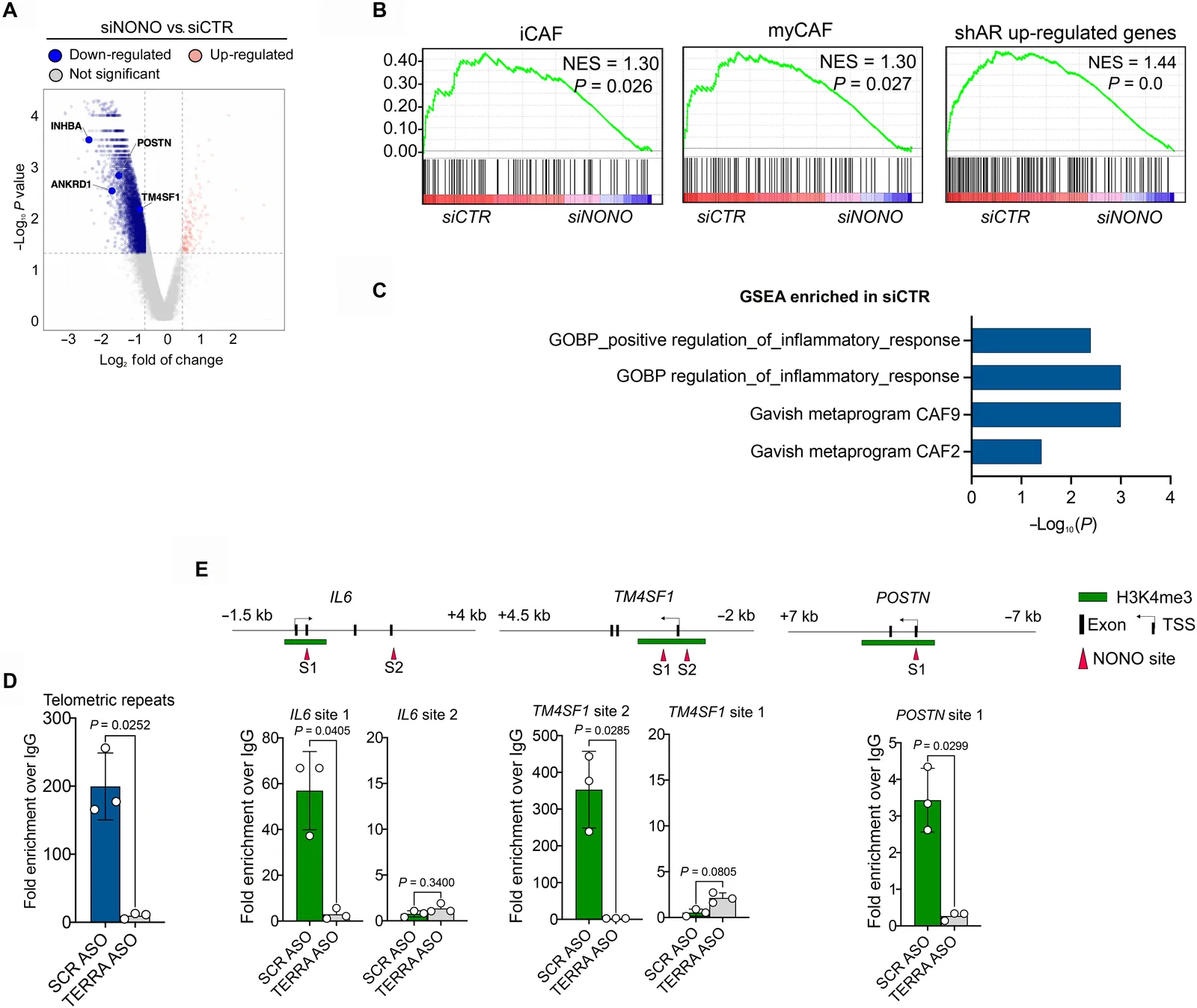
NONO regulates CAF activation in a TERRA-dependent manner. (**A**) Transcriptomic analysis of two CAF strains 72 hours after transfection with control or NONO-targeting siRNAs. The volcano plot shows differentially expressed genes (*P* < 0.05; |log_2_ fold change| ≥ 0.58); selected down-regulated CAF genes are labeled. (**B**) GSEA of inflammatory CAF (iCAF), myofibroblastic CAF (myCAF), and AR-silencing–induced HDF signatures (*16*) in control versus NONO-silenced CAFs. Black bars mark gene positions, and green curves show enrichment scores. Normalized enrichment scores and *P* values are indicated. (**C**) GSEA of selected MSigDB signatures, including CAF9, CAF2, regulation of inflammatory response (GO:0050727), and cytokine production involved in inflammatory response (GO:0002534) (*56*). Bars show −log_10_ *P* values. (**D**) ChIPmentation-qPCR of NONO binding to telomeric repeats in CAFs transfected with scrambled (SCR) or TERRA ASO, using published primers (*57*). (**E**) *IL6*, *TM4SF1*, and *POSTN* promoter regions analyzed. *IL6* and *TM4SF1* primers targeted NONO-bound regions identified from CUT&RUN data (GSE236900) (*40*), *POSTN* primers targeted an H3K4me3-marked promoter region (GSE329794). Bottom: NONO ChIPmentation-qPCR at these loci after SCR or TERRA ASO treatment. Enrichment in (D) and (E) is expressed relative to IgG (*n* = 3 biological replicates). Data are means ± SD; paired *t* test.

GSEA further demonstrated that NONO silencing significantly suppressed transcriptional programs associated with inflammatory and myofibroblastic CAFs, a CAF signature induced by AR loss (*16*), and two CAF metaprograms (Fig. 6, B and C), all of which were similarly reduced by TERRA depletion (Fig. 3, G and H). In line with its broader functional roles, more global GSEA revealed that NONO knockdown affected a wider array of CAF-related gene signatures than TERRA, suggesting additional roles in cellular processes beyond those regulated by TERRA (fig. S7C).

NONO has been shown in other settings to function directly in transcriptional regulation as a coactivator of transcription factors (*7*, *39*). In our system, NONO was expressed at similar levels in CAFs with elevated TERRA and in matched HDF strains, and NONO silencing did not affect TERRA levels (fig. S7D), suggesting that the two act through downstream convergent mechanisms. Previous work in ES cells showed that TERRA associates with chromatin at numerous loci beyond telomeres, including genes such as *IL6*, whose expression is reduced upon TERRA depletion (*5*). Given that TERRA has been proposed to anchor NONO at telomeres (*8*), we asked whether this function extends to nontelomeric genes.

Using a ChIPmentation approach with antibodies validated for efficient NONO immunoprecipitation (fig. S7E), we detected NONO binding at telomeric regions in CAFs, which was strongly reduced by TERRA silencing (Fig. 6D). We then examined CAF effector genes concordantly regulated by NONO and TERRA silencing, with primer placement guided by publicly available NONO chromatin-binding datasets (*40*) (GSE236900). NONO binding was reproducibly detected in independent biological replicates in CAFs to a defined region of the *IL6* gene overlapping the transcription start site (TSS) and the active promoter mark H3K4me3, and this binding was strongly reduced upon TERRA depletion (Fig. 6E). Similar results were obtained for the *TM4SF1* and *POSTN* genes. Together, these findings identify NONO as an important determinant of the transcriptional program underlying CAF activation and indicate that TERRA supports this function by promoting NONO association with regulatory regions of key CAF effector genes.

### Pharmacologic targeting of NONO complex reverses CAF activation, including in human clinical lesions

To assess the translational relevance of our findings, we evaluated the effects of the small-molecule (R)-SKBG-1, which covalently binds to Cys145 in NONO, altering its RNA binding properties and impairing its transcript-processing function (*37*). Relatively to the inactive enantiomer (S)-SKBG-1 or dimethyl sulfoxide (DMSO) control, treatment of CAFs with (R)-SKBG-1 down-regulated key effector and regulatory genes (*POSTN*, *INHBA*, *TM4SF1*, and *ANKRD1*), effectively phenocopying the effects of NONO depletion (Fig. 7A and fig. S8A). (R)-SKBG-1 also prevented the induction of these genes in HDFs following *AR* silencing, mimicking the suppressive effects observed by TERRA knockdown (Fig. 7B and fig. S8B).

**Fig. 7.**
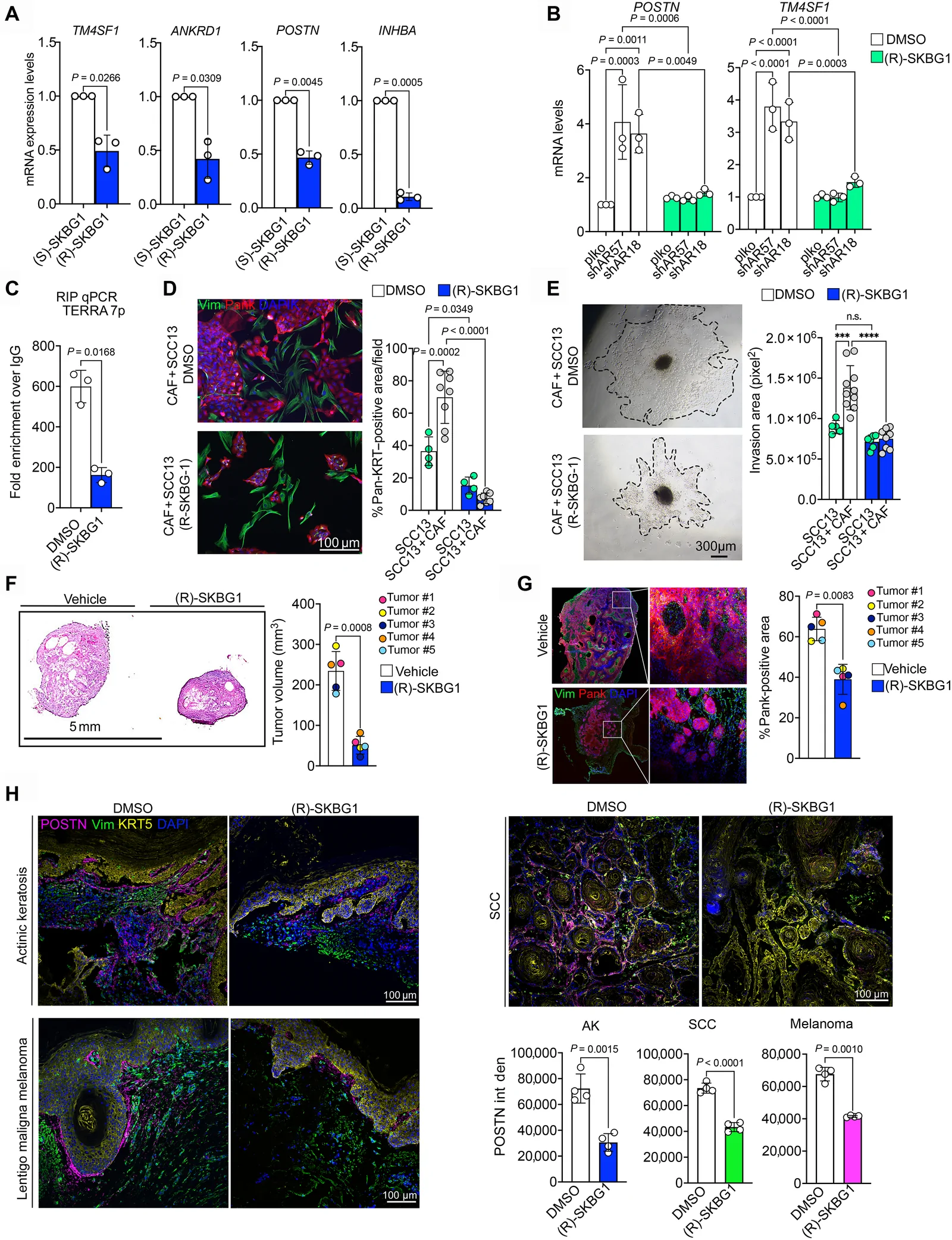
Pharmacological inhibition of NONO disrupts the TERRA-NONO complex and suppresses CAF-driven tumorigenesis. (**A**) RT-qPCR of CAF effector genes after 48-hour treatment with inactive (S)-SKBG1 or active (R)-SKBG1 (5 μM). Expression was normalized to *RPLP0* and is relative to (S)-SKBG1 (*n* = 3 biological replicates). (**B**) RT-qPCR of CAF effector genes in control or AR-silenced HDFs treated with DMSO or (R)-SKBG1 for 48 hours. Expression was normalized to *RPLP0* and is relative to DMSO (*n* = 3 biological replicates). (**C**) NONO RIP–RT-qPCR for Chr7p TERRA in CAFs treated with DMSO or (R)-SKBG1. Results are input-normalized fold enrichment over IgG (*n* = 3 biological replicates). (**D**) SCC13 cells cultured alone or with CAFs treated with DMSO or (R)-SKBG1. Representative IF images show pan-keratin (Pank/Pann-KRT; red) and vimentin (Vim; green); graphs quantify pan-keratin–positive area from three experiments (>4 pooled fields). (**E**) SCC13 spheroid invasion alone or with treated CAFs (>5 spheroids per condition). ***P = 0.0002; ****P < 0.0001; ns, not significant (*P* ≥ 0.05). (**F** and **G**) SCC13 cells admixed with CAFs were injected intradermally into the contralateral flanks of immunocompromised mice. Palpable tumors received daily intratumoral PBS or (R)-SKBG1 (10 μM) for 14 days; tumor volume and pan-keratin (Pank)–positive area were quantified (*n* = 5 pairs). (**H**) Ex vivo actinic keratosis (AK), SCC, and lentigo maligna melanoma biopsies treated with DMSO or (R)-SKBG1 (10 μM, 48 hours) were stained for POSTN, VIMENTIN (Vim), KERATIN 5, and DAPI; POSTN intensity was quantified. Data are means ± SD. Statistics: paired two-tailed *t* test (A, C, F, and G), one-way ANOVA with Šidák’s test (B, D, and E), or unpaired t test with Welch’s correction (H).

(R)-SKBG-1 treatment did not alter overall NONO or TERRA expression levels (fig. S8, C and D) but significantly impaired the ability of NONO to bind TERRA, as determined by RIP in CAFs (Fig. 7C), pointing to TERRA-NONO complex formation as one of its primary targets. (R)-SKBG-1 has previously been shown to inhibit proliferation in various cancer cell types (*37*, *41*, *42*). In our assays, (R)-SKBG-1 reduced SCC cell proliferation in monoculture, with more pronounced effects in coculture with CAFs, where it suppressed CAF-mediated cancer cell expansion (Fig. 7D). In three-dimensional (3D) tumor spheroid models, CAFs promoted SCC invasion, which was markedly attenuated by (R)-SKBG-1 treatment (Fig. 7E), phenocopying the effects of NONO silencing using two independent siRNAs (fig. S8E).

To evaluate the impact of (R)-SKBG-1 in vivo, including its ability to reverse established tumors, SCC cells were coinjected intradermally with CAFs into immunodeficient mice. Once palpable tumors had formed, animals received intratumoral injections of (R)-SKBG-1 or vehicle control for 1 week. As shown in Fig. 7F and fig. S8F, (R)-SKBG-1 treatment resulted in a marked reduction in tumor volume and a significant decrease in cancer cell density (Fig. 7G).

To assess the clinical potential of the findings, we treated freshly excised actinic keratosis, cSCC, and melanoma lesions with (R)-SKBG-1 (fig. S8G). This treatment markedly reduced expression of the CAF marker POSTN across all three lesion types (Fig. 7H), indicating its potential as a potent therapeutic strategy to attenuate CAF activation in patients. Levels of FAP, another established CAF marker (*43*), were similarly decreased (fig. S8H), further supporting the translational potential of targeting this pathway to reverse CAF activation in the clinical setting.

Collectively, these findings show that pharmacologic targeting of NONO with (R)-SKBG-1 effectively reprograms CAFs, phenocopying the effects of TERRA and NONO silencing. The associated reduction in CAF marker expression in freshly excised premalignant and malignant human lesions underscores the translational potential of this approach and identifies (R)-SKBG-1 as a promising candidate for stroma-directed cancer therapy.

## DISCUSSION

Here, we identify a telomere-associated RNA-protein pathway unleashed by loss of AR function that drives fibroblast reprogramming into CAFs. Central to this mechanism is the long noncoding RNA TERRA, which is up-regulated in CAFs and promotes transcriptional reprogramming through interaction with the RNA and DNA binding protein NONO. Our findings support a model in which TERRA and NONO regulate CAF gene expression and CAF activation through a mechanism that is, at least, partly distinct from their canonical functions at telomeres.

Several observations support this conclusion. First, TERRA and NONO exert distinct effects on telomere integrity and CAF gene expression. Although increased TERRA levels influence both telomere stability and CAF transcriptional programs, NONO appears to play opposite roles in these two settings. Second, induction of CAF genes by TERRA does not require an intact DNA damage response as it occurs similarly in HDFs with or without TP53 deletion. Third, high-resolution microscopy shows that TERRA-NONO complexes are present not only at telomeres but also at nontelomeric nuclear sites, predominantly in extranucleolar regions. Fourth, previous studies in embryonic stem cells have shown that TERRA can regulate gene transcription and chromatin organization independently of telomeres through interactions with chromatin regulators (*5*). NONO has likewise been shown in other contexts to act directly in transcriptional regulation as a coactivator of transcription factors (*7*, *39*). TERRA has also been implicated as an anchor for NONO at telomeres (*8*, *44*). Extending these observations, we show that, in CAFs, NONO occupies the regulatory regions of multiple CAF effector genes that are jointly controlled by TERRA and NONO and that this binding is TERRA dependent.

TERRA expression is regulated by a complex interplay of transcription factors, epigenetic modifications, and telomere-binding proteins (*4*, *5*, *21*). Here, we identify AR, a context-dependent transcriptional activator and repressor (*15*), as a negative regulator of this pathway. AR binds subtelomeric regions near TERRA TSSs, and loss of AR, a hallmark of CAF activation (*15*, *16*), leads to increased TERRA levels. Notably, in our previous work using matched HDF and CAF strains (*2*), we did not detect significant differences in bulk telomere length, indicating that the observed increase in TERRA is not attributable to changes in telomere length. At the same time, AR associates with NONO in normal fibroblasts (NFs), whereas in CAFs, AR-NONO complexes are reduced and TERRA-NONO complexes are increased. These observations support a model in which, in normal stromal fibroblasts, AR acts as a dual gatekeeper of the TERRA-NONO axis by repressing TERRA expression while also associating with NONO (Fig. 8). As AR is down-regulated during early CAF activation, this coordinated restraint is released, allowing TERRA levels to rise and favoring enhanced TERRA-NONO complex formation. Whether AR directly limits NONO availability through such a titration mechanism remains to be established.

**Fig. 8.**
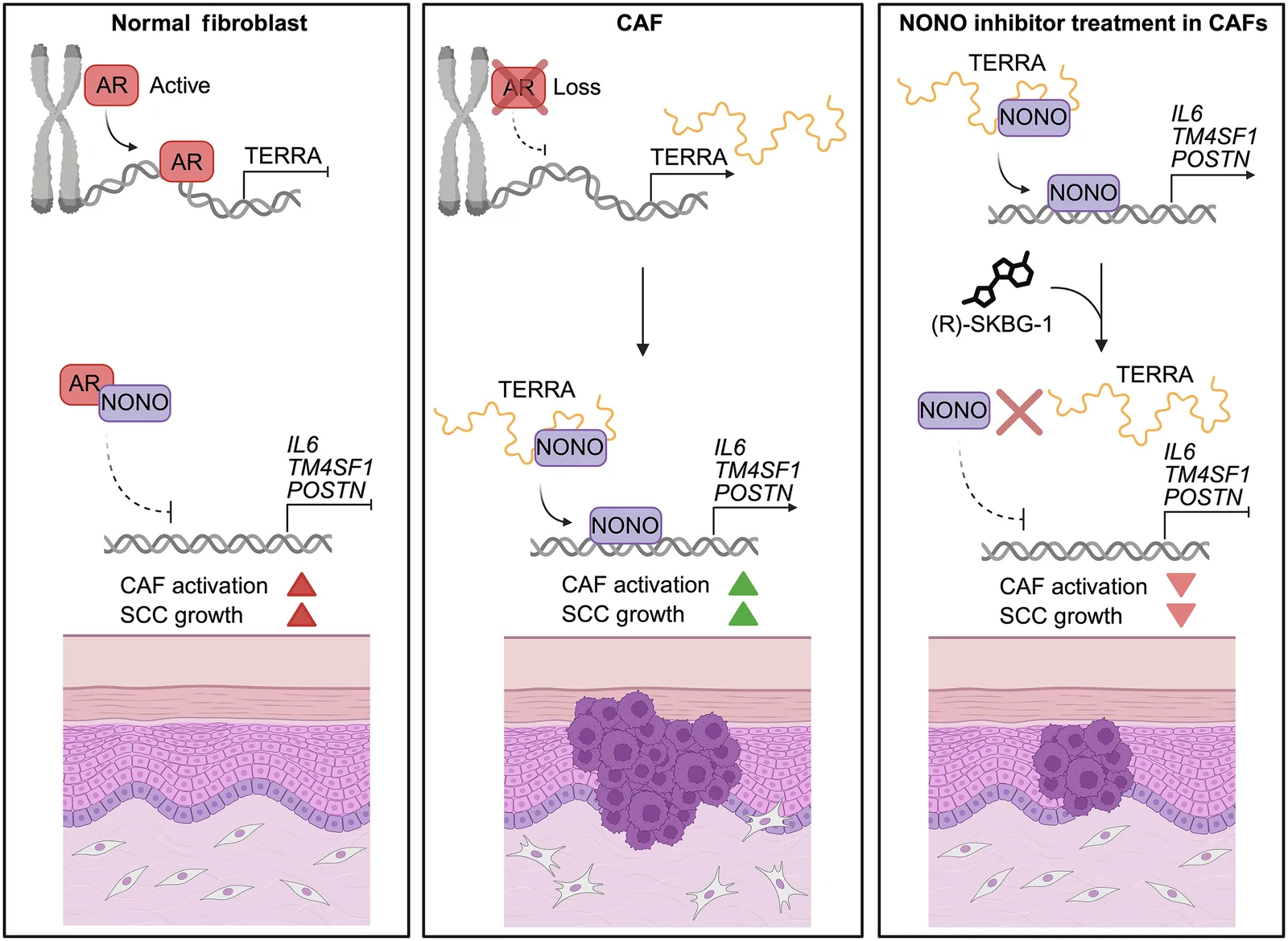
AR-TERRA-NONO axis drives CAF activation and is therapeutically targetable. Schematic model illustrating the regulation and function of the AR-TERRA-NONO pathway in fibroblast reprogramming. In HDFs, active AR represses TERRA transcription at subtelomeric regions, maintaining fibroblast homeostasis and associates with NONO. Upon AR loss, TERRA is up-regulated and associates with the RNA binding protein NONO, promoting its recruitment to the promoters of key CAF effector genes, including *POSTN*, *TM4SF1*, and *IL6*, where NONO directly binds and activates their transcription. This TERRA-NONO–dependent transcriptional program drives fibroblast conversion into tumor-promoting CAFs. Pharmacological inhibition of NONO disrupts the TERRA-NONO complex, leading to suppression of CAF gene expression, restoration of NF features, and reduced SCC tumor-stroma interactions. Created in BioRender. Sol, S. (2026), https://BioRender.com/ap9xyob.

High-resolution microscopy reveals that TERRA and NONO colocalize in CAFs while occupying distinct nuclear compartments. Larger TERRA foci, from which NONO is excluded, localize to nucleolar regions, as indicated by fibrillarin staining. By contrast, smaller TERRA foci, about half of which colocalize with NONO, reside in the nucleoplasm and are distributed equally between telomeric and nontelomeric sites, as defined by TRF2 staining. These observations point to spatially segregated pools of TERRA and NONO, consistent with distinct functions in different nuclear contexts.

At telomeres, NONO localization is TERRA dependent, and NONO exerts a protective function as its silencing enhances the DNA damage response. This is consistent with previous evidence that NONO contributes to telomere integrity by binding TERRA and limiting TERRA-associated telomere dysfunction, including suppression of RNA-DNA hybrid (R-loop) formation (*8*). Notably, NONO typically functions as a heterodimer with SFPQ, which has been shown to regulate TERRA stability at telomeres (*8*), suggesting that additional components of this complex may contribute to the regulation of TERRA function in stromal cells. While the role of SFPQ in CAFs was not directly addressed in the present study, its potential involvement in this pathway represents an important area for future investigation.

Beyond their impact on genome stability, R-loops also have broader roles in transcriptional regulation and gene expression (*45*). A systematic analysis of AR- and TERRA-dependent R-loop dynamics, both at telomeres and genome-wide, should therefore provide further insight into the regulatory axis uncovered here.

Our combined evidence indicates that TERRA-driven transcriptional regulation is not simply a secondary consequence of telomere damage, but rather a direct and functionally distinct process. As a possible underlying mechanism, warranting further investigation, we find that TERRA facilitates NONO recruitment to the regulatory regions of key CAF effector genes, including *IL6*, *TM4SF1*, and *POSTN*. These observations support a model in which TERRA functions as a molecular scaffold that directs NONO to nontelomeric genomic loci, thereby promoting transcriptional programs linked to fibroblast activation. An important next step will be to determine to what extent modulation of TERRA and NONO expression or activity also affects recruitment of other transcription factors and coactivators that regulate these genes, including AP-1 family members and ANKRD1 (*16*).

The translational relevance of these findings is underscored by our demonstration that the small-molecule NONO inhibitor (R)-SKBG-1 (*37*) disrupts the TERRA-NONO interaction and phenocopies the effects of NONO depletion. (R)-SKBG-1 suppresses CAF effector gene expression and impairs stromal-enhanced cancer cell growth in 3D coculture and spheroid invasion models. Previous work showed that (R)-SKBG-1 binds NONO and covalently modifies a specific residue within the hinge region between its two RNA recognition motifs, near residues critical for RNA binding, thereby altering its RNA binding properties (*29*). In the context of CAF activation, we found that (R)-SKBG-1 does not affect TERRA expression or overall NONO protein levels but disrupts TERRA-NONO association and complex formation, consistent with a mode of action targeting RNA-protein interactions rather than gene expression or protein stability. This is unlikely to be its only mechanism of action as (R)-SKBG-1 may also interfere with additional NONO-dependent interactions and exert off-target effects. Nonetheless, treatment of freshly excised human biopsies from actinic keratosis, cSCC, and melanoma with this compound markedly reduced expression of the CAF markers POSTN and FAP, highlighting the therapeutic potential of its utilization for targeting cancer stroma activation.

Together, our findings define a telomere-associated RNA-protein axis that integrates nuclear organization, transcriptional regulation, and stromal cell identity. These mechanisms may have broader implications not only in cancer but also in aging, fibrosis, and chronic inflammatory conditions, where fibroblast activation plays a central role (*46*).

## MATERIALS AND METHODS

### Human subjects

Primary CAFs and matched NFs were isolated from discarded skin specimens of patients with cutaneous SCC (cSCC) undergoing surgery at the Department of Dermatology, Massachusetts General Hospital (MGH; Boston, MA, USA). The study was approved by the Institutional Review Board of MGH (protocol no. 2018P003156), and all patients provided written informed consent for the use of their samples for research purposes.

Shave biopsies of actinic keratosis and lentigo maligna melanoma were obtained from the Department of Dermatology, University Hospital Zürich (Switzerland), with the assistance of the SKINTEGRITY.CH biobank. The use of human samples was approved by the local ethics committee (BASEC_Nr. 2017-00688), and all participants provided written informed consent. All samples used in this study consisted of surplus material from routine surgical procedures.

### Mouse models

NOD.Cg-*Prkdc^scid^ Il2rg^tm1Wjl^*/SzJ mice were obtained from the Jackson Laboratory (stock no. 005557) and maintained under specific pathogen–free conditions in the MGH animal facility. Both male and female mice, 6 to 10 weeks of age at the start of experiments, were used. Mice were housed in individually ventilated cages with a 12-hour light/dark cycle and controlled temperature (20° to 22°C) and humidity (40 to 60%) and provided ad libitum access to autoclaved food and water. All animal experiments were conducted in compliance with the guidelines of the MGH Institutional Animal Care and Use Committee (IACUC) and approved under protocol (2004 N000170).

### Isolation of CAFs from skin

Briefly, after surgical removal of adipose tissue, the biopsies were minced into 1- to 2-mm fragments and digested in Liberase TL (0.25 mg/ml; Roche, catalog no. 05401020001) at 37°C for 40 min. The enzymatic reaction was quenched with fetal bovine serum (FBS), and the cell suspension was filtered through a 70-μm strainer using a syringe. Cells were pelleted by centrifugation at 300*g* and plated in complete Dulbecco’s modified Eagle’s medium (DMEM) containing 10% FBS, 1% penicillin, and 1% streptomycin. Cultures were maintained by splitting at 80 to 90% confluence, with medium changes every 48 hours. Multiple vials were cryopreserved at passage 2, and all experiments were performed using early-passage CAFs (passages 3 to 6). Cell strains were routinely tested for Mycoplasma contamination. BCAFs and MCAFs were previously obtained (*15*).

### Isolation of HDFs

HDFs were isolated from discarded foreskin or abdominoplasty skin samples or were previously established as described (*47*). Multiple vials were cryopreserved at passage 2, and all experiments were performed using early-passage HDFs (passages 3 to 6).

### cSCC cell lines

SCC13 cells were provided by J. Rheinwald (Brigham and Women’s Hospital, Boston, MA, USA). Cells were cultured in DMEM supplemented with 10% FBS and 1% penicillin-streptomycin.

### Lentiviral production and transduction

Lentiviral particle production and infections were performed as previously described (*48*). Briefly, human embryonic kidney 293T cells were seeded to reach ∼60% confluence. After 24 hours, cells were transfected using polyethylenimine (PEI) and the appropriate vector in DMEM supplemented with 10% FBS, without antibiotics. For transfection of a 15-cm dish, 14 μg of total DNA (comprising the vector of interest and packaging plasmids, CMG and VSV-G, in a 4:2:1 ratio) was mixed with 42 μl of PEI (maintaining a 1:3 DNA:PEI ratio), vortexed, and incubated for 15 min at room temperature. During this incubation, the culture medium was replaced with fresh DMEM containing 10% FBS (without antibiotics). The transfection mix was then added to the cells and incubated for 12 hours, after which the medium was replaced with complete DMEM. Viral supernatants were collected 48 hours posttransfection, filtered through 0.45-μm filters, and stored in aliquots at −80°C. Target cells were transduced by overnight incubation with the viral supernatant. The following morning, the medium was replaced with complete DMEM. After 48 hours, antibiotic selection was initiated to enrich for transduced cells.

### shRNA-mediated AR silencing

CAFs and HDFs were transduced with lentiviral shRNA vectors developed by The RNAi Consortium (TRC). HDFs were infected with two independent shRNA constructs targeting the AR: shAR#1 (TRCN0000003718) and shAR#2 (TRCN0000003715), or with the empty pLKO vector as a control. All constructs included a puromycin resistance gene for antibiotic selection.

### TERRA overexpression

HDFs were transduced with a lentiviral vector encoding ∼130 telomeric hexanucleotide repeats consisting of TTAGGG and TTGGGG variant sequences (pLV CMV-TERRA; Addgene, plasmid no. 111375, RRID: Addgene_111375; http://n2t.net/addgene:111375; doi:10.3390/genes7080046). A control vector, pLenti CMV/TO Zeo DEST 644-1 (Addgene, plasmid no. 17249), was used as a negative control. Both vectors contain a zeocin resistance gene for antibiotic selection.

### siRNA-mediated NONO silencing

CAFs were transfected with two independent siRNAs targeting NONO [siNONO#1 (Mission siRNA NM_007363 SASI_Hs02_00343477) and siNONO#2 (Mission siRNA NM_007363 SASI_Hs02_00343479)] or with a nontargeting control siRNA (MISSION siRNA Universal Negative Control #1 SIC001) at the concentration of 30 nM using HiPerFect transfection reagent (QIAGEN), according to the manufacturer’s instructions. Briefly, siRNAs and HiPerFect were mixed in serum-free medium and incubated for 10 min at room temperature to allow complex formation. The siRNA-HiPerFect complexes were then added dropwise to CAFs plated at 60 to 70% confluence in complete medium. Cells were harvested 48 to 72 hours posttransfection for downstream analyses.

### TERRA silencing with antisense oligonucleotides

TERRA was silenced in CAFs using LNA-modified antisense oligonucleotides (ASOs) targeting the telomeric repeat sequence, as previously described (*5*). Briefly, cells were transfected with 100 nM TERRA-specific ASO or a scrambled control ASO using HiPerFect transfection reagent (QIAGEN), according to the manufacturer’s protocol. ASOs and HiPerFect were incubated in serum-free medium for 10 min at room temperature to allow complex formation and then added dropwise to cells plated at 60 to 70% confluence in complete medium. Cells were collected 48 hours posttransfection for downstream analyses.

### CRISPR-P53 HDFs

To generate TP53 knockout fibroblasts, two single guide RNAs targeting TP53 [Integrated DNA Technologies (IDT); 480 pmol each] were complexed with Alt-R S.p. HiFi Cas9 Nuclease (IDT; ∼420 pmol) to form ribonucleoproteins and delivered into early-passage HDFs (HDF18, p2, 8 × 10^5^) by nucleofection (Lonza Human Dermal Fibroblast Nucleofector Kit, Amaxa 2D). The ATM inhibitor KU55933 (10 μM) was added for 10 hours postnucleofection to enhance editing efficiency. Knockout was validated by Western blot (antibodies in table S1).

### Pharmacological treatments with small-molecule inhibitors

HDFs were treated with the AR antagonist UT-155 (MedChemExpress, catalog no. HY-12709) at a concentration of 1 μM for 48 hours in medium supplemented with charcoal-stripped FBS. Activated charcoal for serum stripping was obtained from MilliporeSigma. HDFs were also treated with ARCC4 (Tocris, catalog no. 7254) or its corresponding negative control ARCC4 NC (Tocris, catalog no. 7255) in charcoal-stripped serum under the same conditions. HDFs and CAFs were treated with the selective AR modulator Ostarine (MedChemExpress, catalog no. HY-13273) at 10 μM for 48 hours. Additionally, HDFs and CAFs were treated with the NONO inhibitor (R)-SKBG-1 (MedChemExpress, catalog no. HY-153918) at 5 μM for either 24 or 48 hours.

### EdU incorporation assay

Cell proliferation was assessed using the Click-iT EdU Alexa Fluor 488 Imaging Kit (Invitrogen), following the manufacturer’s instructions. CAFs were seeded onto 19-mm coverslips in six-well plates at ∼40% confluence. The following day, cells were incubated with 10 μM EdU for 4 hours and then fixed with 4% paraformaldehyde (PFA). Stained samples were imaged using a Zeiss fluorescence microscope. Quantification of EdU-positive cells was performed using ImageJ software, measuring the number of EdU-positive nuclei per field.

### Coculture assays

To assess tumor cell expansion in fibroblast–cancer cell coculture, cells were plated in eight-well chamber slides precoated with Matrigel (BD Biosciences). Each well was coated with 100 μl of Matrigel diluted 1:10 in cold medium and incubated at 37°C for 30 min to allow polymerization. HDFs or CAFs were mixed with SCC13 cancer cells at a 1:1 ratio (1000 fibroblasts and 1000 cancer cells per well) and then seeded onto the coated chambers. Tumor cell expansion was evaluated 5 days postseeding by immunofluorescence staining for Pan-keratin (1:300) to label epithelial cancer cells, vimentin (1:300) (table S1) to identify fibroblasts, and 4′,6-diamidino-2-phenylindole (DAPI; 1:1000) to visualize nuclei. Image acquisition was performed using a fluorescence microscope, and quantification of tumor expansion was conducted using ImageJ software.

### Multicellular spheroid invasion assay

Multicellular spheroids were generated from subconfluent cultures using the hanging-drop method (*49*). Briefly, CAFs and/or SCC13 cells were resuspended in complete DMEM supplemented with 10% FBS, methylcellulose (4.8 mg/ml; Sigma-Aldrich, catalog no. M6385), and bovine dermis collagen I solution (10 mg/ml; PureCol, Advanced BioMatrix Inc., catalog no. 5005) at a final concentration of 3000 cells per 30-μl drop. Drops were placed on the inner side of a 10-cm dish lid and incubated in suspension for 24 hours in an incubator at 37°C and 5% CO_2_ to allow spheroid formation. The preformed spheroids were then embedded in 3D collagen matrices. The collagen solution was prepared using mid-density rat-tail collagen (final concentration, 5 mg/ml). First, a total volume of 1400 ml was obtained by adding 112 ml of 10× phosphate-buffered saline (PBS), 13.89 ml of 1 N NaOH, 299.7 ml of 80 mM Hepes in Milli-Q water, and 632.3 ml of nonpepsinized rat-tail collagen type I stock solution (11.07 mg/ml; Corning, catalog no. 354249) to the mixture. Spheroids composed of CAFs and SCC13 cells (mixed or separate) were washed twice with PBS (5 min each), resuspended in 300 ml of medium, and mixed with collagen solution. Homogeneous collagen solution (150 ml) was transferred into an eight-chamber slide (at room temperature) with four to five spheroids per well. The spheroids were positioned at the interface between collagen and plastic before final polymerization. Invasion has been detected by bright-field microscopy 2 days postembedding. Image analysis was performed using Fiji/ImageJ. The invasion area was quantified as the 2D area surrounding the spheroid, calculated by subtracting the area of the spheroid core from the total invaded region. Spheroids that fused or were located at the gel edge were excluded from analysis.

### Immunofluorescence staining and quantification

For immunofluorescence staining of cultured cells, coverslips were first washed with cold PBS and then fixed with 4% formaldehyde in PBS for 10 min at room temperature. Cells were permeabilized with 0.5% Triton X-100 in PBS for 15 min and subsequently washed with PBS. Blocking was performed using 2% BSA in PBS for 30 min. Cells were then incubated overnight at 4°C with primary antibodies diluted in blocking solution. The following day, coverslips were washed three times with PBS and incubated for 1 hour at room temperature with Alexa Fluor–conjugated secondary antibodies (Alexa Fluor 488, Alexa Fluor 568, or Alexa Fluor 647; 1:1000 dilution; Thermo Fisher Scientific). After final washes, coverslips were mounted using Dako Fluorescence Mounting Medium (Agilent Technologies). Images were acquired using a Zeiss LSM700 confocal microscope. Quantification of fluorescence signals was performed using Fiji/ImageJ software. For tissue sections, tumors were embedded in OCT compound (Tissue-Tek), snap frozen at −80°C, and cryosectioned into 7- to 8-μm sections using a cryostat. Sections were air-dried for 30 min at room temperature and fixed in 4% PFA for 20 min. The staining procedure was then carried out as described for cultured cells.

### TERRA RNA-FISH

Detection of TERRA RNA foci in HDFs and CAFs was performed using a Stellaris RNA-FISH protocol with a Cy3-labeled TelC probe (PNA bio F1002, TelC-Cy3) complementary to the telomeric UUAGGG repeat. Cells were seeded on coverslips and fixed in 4% PFA in PBS for 10 min at room temperature, followed by permeabilization in 70% ethanol at −20°C for at least 1 hour. After fixation, coverslips were incubated with RNase H (NEB, 5 U in 1× RNase H buffer) for 3 hours at 37°C as previously described (*17*). Hybridization was carried out overnight at 37°C in Stellaris Hybridization Buffer (Biosearch Technologies) supplemented with 125 nM TelC-555 probe in a humidified chamber. The following day, coverslips were washed twice for 30 min at 37°C in Stellaris Wash Buffer A, counterstained with DAPI, and briefly rinsed in Wash Buffer B. Coverslips were mounted using ProLong Antifade Mounting Medium and imaged using a Nikon CSU-W1 SoRa spinning disk confocal microscope. Quantification of nuclear TERRA foci was performed using Fiji/ImageJ software.

### Immuno-RNA-FISH for NONO and TERRA on cells

Combined immunofluorescence and RNA-FISH (immuno-RNA-FISH) was performed on CAFs to detect the colocalization of the NONO protein and TERRA RNA, using a sequential protocol adapted from the Stellaris RNA FISH guidelines. CAFs were seeded on glass coverslips and fixed in 4% PFA in PBS for 10 min at room temperature, followed by permeabilization with 0.5% Triton X-100 in PBS for 10 min. After washing, cells were blocked in 2% BSA in PBS for 30 min and incubated overnight at 4°C with a rabbit anti-NONO antibody (table S1) in blocking solution. The next day, cells were washed with PBS and incubated for 1 hour at room temperature with Alexa Fluor–conjugated secondary antibodies (1:1500, Thermo Fisher Scientific). After staining, cells were postfixed again with 4% PFA for 10 min to preserve antibody-antigen complexes during RNA-FISH. Following postfixation, cells were briefly washed in Stellaris Wash Buffer A. Hybridization was performed overnight at 37°C in Stellaris Hybridization Buffer (Biosearch Technologies) containing 125 nM Cy3-labeled TelC probe (PNA bio F1002, TelC-Cy3, complementary to UUAGGG repeats). The following day, cells were washed twice in Stellaris Wash Buffer A for 30 min at 37°C, counterstained with DAPI, and briefly rinsed in Wash Buffer B. Coverslips were mounted using ProLong Antifade Mounting Medium and imaged using a Nikon CSU-W1 SoRa spinning disk confocal microscope. Colocalization of TERRA RNA foci and NONO protein was analyzed using Fiji/ImageJ software.

### Immuno-RNA-FISH for TERRA and vimentin in human tissue sections

Immuno-RNA-FISH was performed on frozen tissue sections from human SCC and matched adjacent skin and on a frozen tissue array of lung (BioChain, T6235152-5, lot. C708031) to visualize TERRA RNA and vimentin protein. Sections were air-dried for 30 min at room temperature, followed by fixation in 4% PFA in PBS for 10 min. After washing in PBS, sections were permeabilized in 0.5% Triton X-100 in PBS for 10 min and then blocked in 5% normal donkey serum in PBS for 1 hour at room temperature. Sections were incubated overnight at 4°C with a mouse anti-vimentin antibody (Vim; 1:300; Abcam, ab137321) diluted in blocking solution. The next day, sections were washed with PBS and incubated with an Alexa Fluor–conjugated donkey anti-mouse secondary antibody (1:1000; Thermo Fisher Scientific) for 1 hour at room temperature. Following immunostaining, slides were postfixed in 4% PFA for 10 min and then briefly incubated with Stellaris Wash Buffer A. Hybridization was carried out overnight at 37°C in Stellaris Hybridization Buffer (Biosearch Technologies) containing 125 nM TelC-Cy3 probe (PNA bio F1002, TelC-Cy3, complementary to UUAGGG repeats). Posthybridization, sections were washed twice for 30 min at 37°C in Stellaris Wash Buffer A, counterstained with DAPI, and briefly rinsed in Wash Buffer B. Slides were mounted using ProLong Antifade Mounting Medium and imaged using a Nikon CSU-W1 SoRa spinning disk confocal microscope. The percentage of vimentin-positive cells in which at least one TERRA focus has been detected was analyzed using Fiji/ImageJ software.

### Proximity ligation assay

PLAs were performed using the Duolink In Situ Red Starter Kit (Sigma-Aldrich, catalog no. DUO92101) according to the manufacturer’s instructions. Cells were seeded on glass coverslips in a 24-well plate and fixed in cold 4% PFA for 15 min at room temperature, followed by three washes with PBS. After fixation, cells were permeabilized with 0.1% Triton X-100 in PBS for 15 min at room temperature and then incubated with Duolink Blocking Solution for 1 hour at 37°C in a humidified chamber. Following blocking, cells were incubated overnight at 4°C with primary antibodies diluted in Duolink Antibody Diluent. The next day, cells were washed and incubated with the appropriate Duolink PLA probes (PLUS and MINUS) for 1 hour at 37°C in a humidified chamber. After washing three times with Buffer A (provided in the kit), cells were incubated with the ligation mix for 1 hour at 37°C, followed by another three washes in Buffer A. Amplification was then performed by incubating the cells with the amplification mix for 140 min at 37°C in a dark, humidified chamber. After amplification, cells were washed twice in Buffer B for 10 min each, followed by a brief wash in 0.01× Buffer B for 1 min. Coverslips were mounted with Duolink In Situ Mounting Medium containing DAPI. Images were acquired using a Zeiss microscope, and PLA signals were quantified using ImageJ software by performing fluorescent particle analysis to count the number of PLA puncta per nucleus per field.

Antibodies used were anti-rabbit NONO (1:200) and anti-mouse AR (1:100) (table S1).

### Immunoblotting

Cells were lysed directly in NuPAGE LDS Sample Buffer (4×; Thermo Fisher Scientific, NP0007) supplemented with 1% β-mercaptoethanol and protease/phosphatase inhibitors (Thermo Fisher Scientific). Lysates were collected and briefly sonicated and then boiled at 95°C for 5 min to ensure complete protein denaturation. An equal volume of protein was loaded and resolved on 4 to 12% Bis-Tris NuPAGE gels (Thermo Fisher Scientific) using Mops SDS Running Buffer and subsequently transferred to polyvinylidene difluoride membranes. Membranes were blocked in 5% nonfat dry milk in tris-buffered saline with 0.1% Tween 20 for 1 hour at room temperature and incubated overnight at 4°C with primary antibodies diluted in blocking buffer (table S1). After washing, membranes were incubated with horseradish peroxidase–conjugated secondary antibodies for 1 hour at room temperature. Protein bands were visualized using enhanced chemiluminescence (ECL; Thermo Fisher Scientific) and imaged with Azure Biosystem Chemidoc Imaging System or detected on Fuji Medical x-ray films (Fujifilm).

### Gene expression analysis by real-time qPCR

Total RNA was extracted using the Direct-zol RNA Miniprep Kit (Zymo Research, catalog no. R2050) according to the manufacturer’s instructions, including on-column deoxyribonuclease (DNase I) treatment to eliminate genomic DNA contamination. RNA concentration and purity were assessed using a NanoDrop spectrophotometer (Thermo Fisher Scientific). For cDNA synthesis, 500 ng of total RNA was reverse transcribed using the iScript Reverse Transcription Supermix (Bio-Rad, catalog no. 1708840) in a 20-μl reaction, following the manufacturer’s protocol. Real-time qPCR was performed on an Applied Biosystems QuantStudio 5 Real-Time PCR System using Applied Biosystems SYBR Green PCR Master Mix (catalog no. 4309155). Gene expression was analyzed using the comparative Ct (ΔΔCt) method. All reactions were run in technical triplicate, and expression levels were normalized to the endogenous control gene RPLP0. Primers sequences are listed in table S2.

### RNA extraction, DNase treatment, reverse transcription, and RT-qPCR for TERRA

Total RNA was extracted from cultured cells using the Direct-zol RNA Miniprep Kit (Zymo Research, catalog no. R2050), following the manufacturer’s instructions. Cells were lysed in TRIzol reagent and processed through Zymo-Spin columns, including successive on-column DNase I treatment to ensure thorough removal of genomic DNA. RNA was eluted in RNase-free water and quantified using a NanoDrop spectrophotometer (Thermo Fisher Scientific). Only samples with optical density at 260 nm (OD_260_)/OD_280_ ≥ 1.8 were used for downstream applications.

Because only ∼7% of TERRA transcripts are polyadenylated, most do not undergo full polyA maturation (*50*). To capture the broad population of TERRA transcripts, reverse transcription was performed using a TERRA-specific primer composed of telomeric repeats (table S2). To enable normalization, a glyceraldehyde-3-phosphate dehydrogenase (GAPDH) gene-specific reverse primer was included in the same reverse transcription reaction (table S2). Reverse transcription was carried out using 1 μg of total DNase-treated RNA (as previously described), combined with the iScript Reverse Transcription Supermix (Bio-Rad, catalog no. 1708840) in a 20-μl final volume and following the manufacturer’s protocol. A no-reverse transcriptase (−RT) control was included to confirm the absence of genomic DNA contamination. The resulting cDNA was used for RT-qPCR using specific primer pairs targeting TERRA transcripts from subtelomeric regions (table S2) and GAPDH as the internal normalization control. All reactions were performed in technical triplicate and analyzed using the ΔΔCt method.

### RIP for TERRA enrichment in CAFs and HDFs

RIP was performed in CAFs or HDFs to assess the interaction between the RNA binding protein NONO and TERRA transcripts originating from chromosome 7p. Approximately 4 million cells per condition were harvested, washed with cold PBS, and lysed in RIP lysis buffer [10 mM Hepes (pH 7.4), 5 mM MgCl_2_,100 mM KCl, 2 mM EDTA (pH 8), 0.5% NP-40, and 0.5 mM dithiothreitol (DTT)], supplemented with RNase and protease inhibitors. Lysates were cleared by centrifugation at 12,000*g* for 10 min at 4°C, precleared with anti-rabbit immunoglobulin G (IgG) magnetic beads (Invitrogen, catalog no. 11204D) for 1 hour at 4°C, and incubated for 1 hour at RT with 5 μg of anti-NONO antibody (table S1) or 5 μg of normal rabbit IgG (control). Immune complexes were captured by incubation with anti-rabbit IgG magnetic beads for 3 hours at 4°C under rotation. Beads were washed extensively with high-salt wash buffer [100 mM NaCl, 10 mM tris-HCl (pH 7.4), 0.5% SDS, and 1 mM EDTA (pH 8)] containing RNase and protease inhibitors. RNA was extracted from immunoprecipitates using TRIzol reagent (Invitrogen), precipitated with ethanol, and reverse transcribed as previously described. RT-qPCR was performed using primers specific for TERRA transcripts from chromosome 7p. Amplification of RPLP0 mRNA was included as a negative control for NONO binding. A 10% input sample was processed in parallel and used for normalization. Enrichment was calculated using the percentage of input method: %Input = 100 × 2(CtInput − CtIP).

### ChIPmentation q-PCR

ChIPmentation coupled with qPCR analysis was performed using antibodies against NONO (5 μg) and AR (10 μg), along with nonimmune IgG controls and input chromatin. Immunoprecipitations were conducted using 2 × 10^6^ CAFs for NONO and 2 × 10^7^ HDFs cells for AR. Briefly, cells were cross-linked, nuclei were isolated, and chromatin was fragmented by sonication. ChIP was carried out following established protocols (*16*, *51*). Before elution, bead-bound chromatin was subjected to tagmentation using Tn5 transposase (Nextera DNA Sample Preparation Kit, Illumina). Tagmented chromatin was then de–cross-linked and treated with proteinase K (Roche). Equal amounts of purified DNA (5 ng) were amplified using tag-specific primers. The amplified products were diluted (1:5), and 2 μl was used as a template for qPCR to quantify enrichment at the indicated genomic regions. Primer sequences and antibody details are provided in tables S1 and S2.

### Identification of TERRA-interacting proteins by iDRIP

iDRIP was performed in CAFs following the protocol by Chu *et al*. (*5*), with minor modifications. Approximately 10 million cells per condition were UV–cross-linked at 254 nm (400 mJ/cm^2^) to preserve endogenous RNA-protein interactions. Cells were lysed in iDRIP lysis buffer [20 mM tris-HCl (pH 7.5), 150 mM NaCl, 1 mM EDTA, 1% NP-40, 0.1% SDS, 0.5% sodium deoxycholate, and 1 mM DTT], supplemented with protease and RNase inhibitors. Lysates were treated with DNase and low concentrations of RNase I (Thermo Fisher Scientific) to fragment RNA while preserving RNA-protein cross-links. Pulldown was carried out using a biotinylated antisense DNA probe complementary to the UUAGGG repeat region of TERRA (TERRA-AS), or a biotinylated sense probe (TERRA-SENSE) was used in parallel as a negative control, hybridized at 37°C for 2 hours. Streptavidin-conjugated magnetic beads were used to isolate probe-bound complexes and sent to the Harvard Taplin Biological Mass Spectrometry facility for liquid chromatography–mass spectrometry (LC-MS/MS).

### Bead digestion analysis by LC-MS/MS

Beads were washed at least five times with 100 μl of 50 mM ammonium bicarbonate, then 5 μl of modified sequencing-grade trypsin (200 ng/μl; Promega, Madison, WI) was spiked in, and the samples were placed in a 37°C room overnight. The samples were then centrifuged or placed on a magnetic plate if magnetic beads were used, and the liquid was removed. The extracts were then dried in a speed-vac (∼1 hour). Samples were then resuspended in 50 μl of HPLC solvent A (2.5% acetonitrile and 0.1% formic acid) and desalted by STAGE tip (*52*). On the day of analysis, the samples were reconstituted in 10 μl of HPLC solvent A. A nanoscale reverse-phase HPLC capillary column was created by packing 2.6-μm C18 spherical silica beads into a fused silica capillary (100-μm inner diameter by ∼30-cm length) with a flame-drawn tip (*53*). After equilibrating the column, each sample was loaded via a Famos auto sampler (LC Packings, San Francisco, CA) onto the column. A gradient was formed, and peptides were eluted with increasing concentrations of solvent B (97.5% acetonitrile and 0.1% formic acid). As peptides eluted, they were subjected to electrospray ionization and then entered into a Velos Orbitrap Elite ion-trap mass spectrometer (Thermo Fisher Scientific, Waltham, MA). Peptides were detected, isolated, and fragmented to produce a tandem mass spectrum of specific fragment ions for each peptide. Peptide sequences (and hence protein identity) were determined by matching protein databases with the acquired fragmentation pattern by the software program, Sequest (Thermo Fisher Scientific, Waltham, MA) (*54*). All databases include a reversed version of all the sequences, and the data were filtered to a 1 and 2% peptide false discovery rate.

### Transcriptomic analysis

Total RNA was extracted and purified using the Direct-zol RNA Miniprep Kit (Zymo Research). RNA quality was assessed to ensure OD_260_/OD_280_ ≥ 1.8 and RNA integrity number ≥ 8. The GeneChip WT PLUS Reagent Kit (Thermo Fisher Scientific) was used for sample preparation, and hybridization was performed on the human Clariom D Arrays (Thermo Fisher Scientific). Microarray experiments and data acquisition were carried out at the Institute of Genetics and Genomics of Geneva (iGE3). Data processing and analysis were performed using the Transcriptome Analysis Console software (Thermo Fisher Scientific). Global transcriptomic changes were further analyzed using GSEA with curated gene signatures from the MSigDB v7.2 database (www.gsea-msigdb.org/gsea/msigdb).

### Intradermal back injection in mice

Intradermal tumorigenicity assays were performed in 6- to 10-week-old male and female NOD/SCID mice (the Jackson Laboratory). A total of 2.5 × 10^5^ SCC13 cells were admixed with an equal number of HDFs with or without TERRA overexpression or with TERRA-silenced CAFs. After centrifugation, cell pellets were resuspended in 100 μl of Matrigel (BD Biosciences) and injected intradermally into the left and right flanks of the mouse back. Mice were euthanized 10 days postinjection for tissue analysis. Tumor volume was calculated using the formula: *V* (*L* × *W*^2^)/2, where *V* is volume, *W* is width, and *L* is length. Tumors did not exceed the maximum dimension permitted by the approved protocol (20 mm in any direction). For intratumoral delivery of (R)-SKBG-1, a total of 2.5 × 10^5^ SCC13 cells were admixed with an equal number of CAFs. Upon detection of palpable lesions (∼5 mm in diameter) 7 days postinjection, (R)-SKBG-1 (10 μM diluted in PBS) or vehicle (PBS) was administered intratumorally into paired lesions once daily for 14 days. All animal procedures were conducted in accordance with MGH IACUC–approved protocol (2004 N000170).

### Ex vivo biopsy culture and treatment

Freshly collected surplus biopsies from cSCC, actinic keratosis, and lentigo maligna melanoma were bisected and immediately used for ex vivo culture as described before (*55*). Briefly, the biopsies were placed in cell culture inserts (Millipore, catalog no. Z352985). Inserts were positioned in six-well plates containing 1.5 ml of coculture medium in the lower compartment only. Biopsies were treated with either DMSO (vehicle control) or (R)-SKBG1 (10 μM) and maintained for 2 days, with daily medium replacement. Following the treatment period, hematoxylin and eosin staining was performed to assess tissue integrity.

The coculture medium consisted of 3 parts DMEM (Gibco, catalog no. 41966052) and 1 part Ham’s F-12 Nutrient Mix (Gibco, catalog no. 11765054), supplemented with 10% heat-inactivated FBS (Gibco, catalog no. 1050), normocin (0.1 mg/ml; Invivogen, catalog no. ant-nr-1), adenine (21.8 μg/ml; Sigma-Aldrich, catalog no. A2786), apotransferrin (5.45 μg/ml; Sigma-Aldrich, catalog no. T1147), 2.18 nM triiodothyronine (Sigma-Aldrich, catalog no. T6397), hydrocortisone (0.44 μg/ml; Sigma-Aldrich, catalog no. H0888), 0.11 nM cholera toxin (Sigma-Aldrich, catalog no. C8052), insulin (5.50 μg/ml; Sigma-Aldrich, catalog no. I6634), and epidermal growth factor (0.01 μg/ml; Sigma-Aldrich, catalog no. E4127).

### Statistical significance

All statistical analyses were performed using GraphPad Prism version 11 (GraphPad Software, Inc.). As indicated in the figure legends, data are presented as means ± SD. Detailed descriptions of the statistical methods applied to each experiment are provided in the corresponding legends. Unless otherwise noted, two-tailed Student’s *t* tests were used to compare two groups. For comparisons involving more than two groups, one-way analysis of variance (ANOVA) was performed, followed by Šidák’s, Tukey’s, or Dunnett’s multiple comparisons tests, as appropriate.

### Language editing

Large language models, including ChatGPT (OpenAI), were used to assist in improving the clarity and fluency of English in the manuscript text. These tools were not used for data analysis, interpretation, or generation of scientific content.
