# Supplementary material for: A TERRA-NONO axis drives fibroblast reprogramming in cancer
Source: Sci Adv. 2026 Oct 2;12(40):eaed3358. doi: 10.1126/sciadv.aed3358 (PMC13641401; doi:10.1126/sciadv.aed3358)
Supplement: Supplementary file 1 [file sciadv.aed3358_sm.pdf]

Science Advances
AAAS

Supplementary Materials for

A TERRA-NONO axis drives fibroblast reprogramming in cancer

Emery Di Cicco *et al.*

Corresponding author: Gian Paolo Dotto, gdotto@mgh.harvard.edu

Sci. Adv. **12**, eaed3358 (2026)
DOI: 10.1126/sciadv.aed3358

The PDF file includes:

Figs. S1 to S8
Tables S1 and S2
Legend for data file S1

Other Supplementary Material for this manuscript includes the following:

Data file S1

Supplementary material

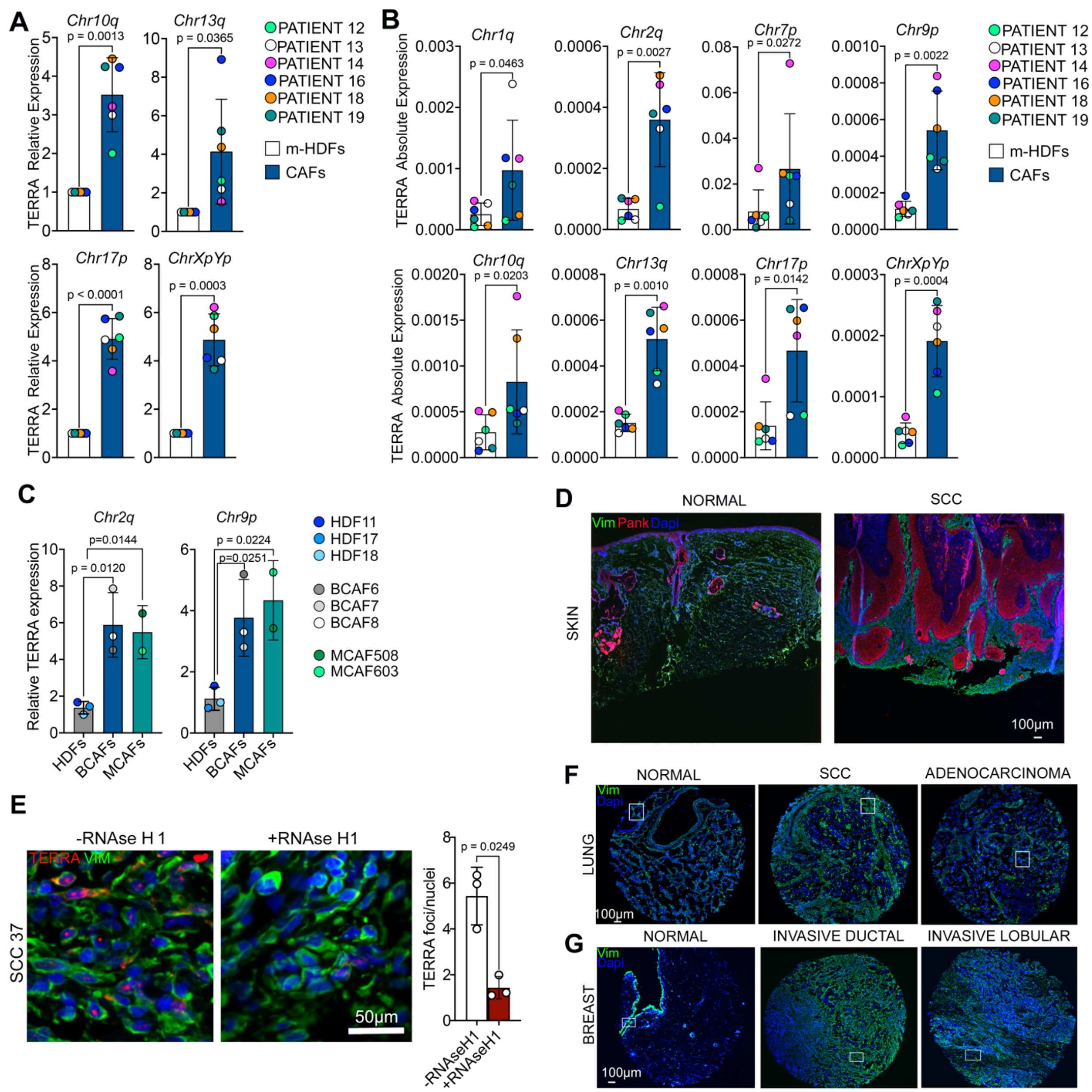

fig. S1 Terra is highly expressed in CAFs vs matched fibroblasts. (A) Chromosome-specific TERRA expression in multiple CAF strains isolated from skin SCC lesions (CAFs) compared to HDFs from adjacent unaffected skin (HDFs) from the same patients as in Fig.1a, as determined by RT-qPCR using specific primers. Expression is shown as fold change in CAFs versus matched HDFs, normalized to GAPDH. n = 6 (strains). Mean ± SD; two-tailed paired t-test. **(B)** Absolute TERRA expression in multiple CAF compared to dermal fibroblasts from adjacent unaffected skin (HDFs) of the same patients, assessed by RT-qPCR as in Figure 1a. mean ± SD. Two-tailed

paired *t*-test. **(C)** RT-qPCR analysis of chromosome-specific TERRA expression in CAFs isolated from BCC lesions (BCAFs) (BCAF6, BCAF7, BCAF8) and melanomas (MCAF508, MCAF603) compared to three age-matched reference HDFs (HDF11, HDF17, HDF18) normalized to GAPDH. n = 3 strains (BCAFs) n=2 strains (MCAFs). Mean ± SD. One-way ANOVA with Dunnett's multiple comparisons test. **(D)** Representative immunofluorescence images of normal skin and squamous cell carcinoma (SCC) sections stained for vimentin (Vim; green), pan-keratin (Pank; red), and DAPI (blue) to distinguish stromal and epithelial compartments. These samples correspond to SCC lesions subsequently analyzed by combined TERRA RNA FISH and immunofluorescence in parallel sections. (**E**) Representative images and quantification of combined immunofluorescence (IF) for vimentin (stromal cell marker) and RNA FISH using a TERRA probe in SCC lesions, with or without RNase H treatment (5 U) as a specificity control. Quantification shows the number of TERRA foci per nucleus. Quantification was performed per nucleus, with >100 cells analyzed per replicate and averaged to generate one data point (n = 3 biological replicates). Mean ± SD. Paired t-test. **(F)** Low magnification images of normal lung tissue, lung squamous cell carcinoma (SCC), and lung adenocarcinoma from a tissue array stained with anti-vimentin antibody to identify stromal cells and TERRA RNA FISH. The high magnification area shown in Fig.1E is indicated by a square. **(G)** Low magnification images of normal breast tissue, invasive ductal carcinoma, and invasive lobular carcinoma from a tissue array stained with anti-vimentin antibody to identify stromal cells and TERRA RNA FISH. The high magnification area shown in Fig.1F is indicated by a square.

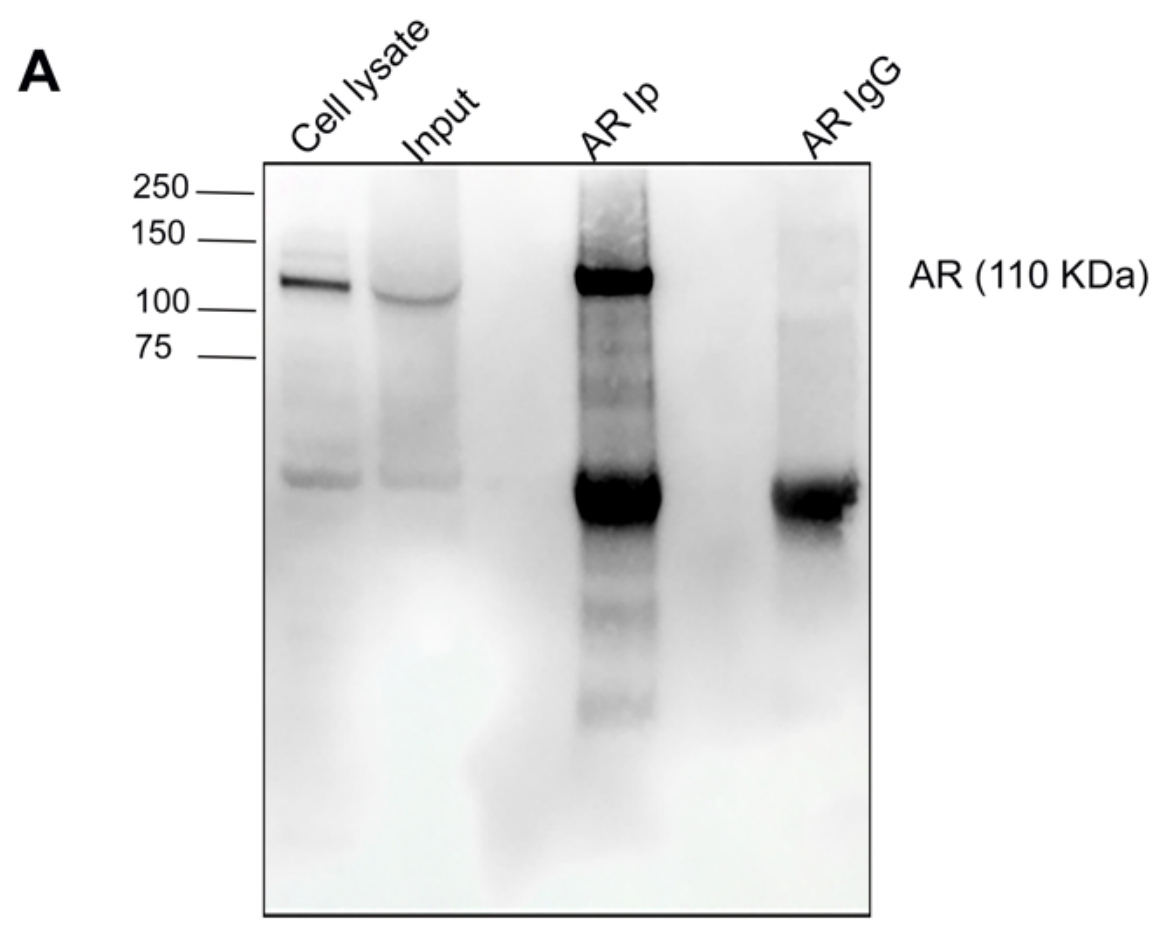

fig. S2 Validation of AR ChIPmentation by immunoprecipitation in HDFs. Representative Western blot showing androgen receptor (AR) pulldown from human dermal fibroblasts (HDFs) used for ChIPmentation experiments. Cell lysate and input fractions are shown alongside immunoprecipitation with anti-AR antibody (AR IP) and IgG control (AR IgG). A specific band corresponding to AR (~110 kDa) is strongly enriched in the AR IP compared to IgG, confirming efficient and specific AR immunoprecipitation for downstream ChIPmentation analyses.

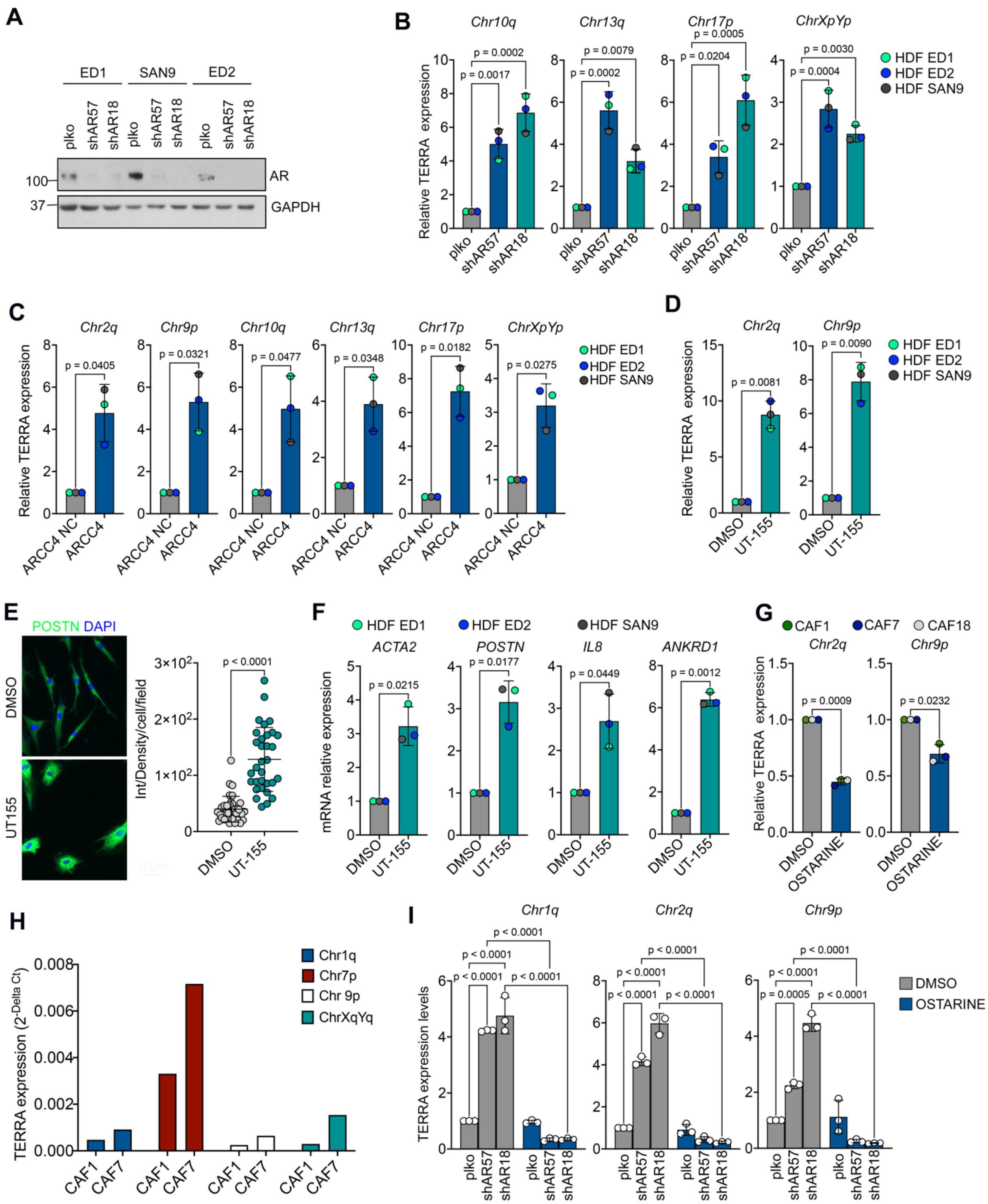

A
ED1
SAN9
ED2
plko
shAR57
shAR18
100
37
AR
GAPDH
B
Chr10q
Chr13q
Chr17p
ChrXpYp
Relative TERRA expression
p = 0.0002
p = 0.0017
p = 0.0079
p = 0.0002
p = 0.0005
p = 0.0204
p = 0.0030
p = 0.0004
HDF ED1
HDF ED2
HDF SAN9
C
Chr2q
Chr9p
Chr10q
Chr13q
Chr17p
ChrXpYp
p = 0.0405
p = 0.0321
p = 0.0477
p = 0.0348
p = 0.0182
p = 0.0275
ARCC4 NC
ARCC4
D
Chr2q
Chr9p
p = 0.0081
p = 0.0090
DMSO
UT-155
E
POSTN DAPI
DMSO
UT155
Int/Density/cell/field
p < 0.0001
F
ACTA2
POSTN
IL8
ANKRD1
mRNA relative expression
p = 0.0215
p = 0.0177
p = 0.0449
p = 0.0012
G
CAF1
CAF7
CAF18
Chr2q
Chr9p
p = 0.0009
p = 0.0232
DMSO
OSTARINE
H
TERRA expression (2^-Delta Ct)
Chr1q
Chr7p
Chr 9p
ChrXqYq
CAF1
CAF7
I
Chr1q
Chr2q
Chr9p
TERRA expression levels
p < 0.0001
p = 0.0005
DMSO
OSTARINE

fig. S3. TERRA Transcription Is Repressed by AR in HDFs (A) Immunoblot analysis with anti-AR and GAPDH antibodies of three HDFs strains following AR silencing by infection with two different shRNAs silencing lentiviruses (shAR#57, shAR#18) versus control (plko). **(B)**Chromosome-specific TERRA expression in multiple HDF strains following AR silencing by infection with two different shRNAs silencing lentiviruses (shAR#57, shAR#18) versus control (plko), measured by RT-qPCR with specific primers. Expression is shown as fold change relative to control, normalized to GAPDH. n = 3 HDF strains. Data represent mean ± SD. One-way ANOVA with Dunnett's multiple comparisons test. **(C)** Chromosome-specific TERRA expression (in three HDF strains treated with ARCC4 (1 μM, 48 h) versus ARCC4-negative control (ARCC4 NC) as in the previous panel. Expression is shown as fold change relative to ARCC4 NC, normalized to GAPDH. n = 3 strains. Mean ± SD. Paired two-tailed t-test. **(D)** Chromosome-specific TERRA expression in three HDF strains treated with UT-155 (1 μM, 48 h) versus DMSO control, expressed as fold change relative to DMSO. n = 3 strains. Mean ± SD. Paired two-tailed t-test. **(E)** Representative images and quantification of immunofluorescence with antibody against periostin (POSTN) (green) in HDFs treated with UT-155 versus DMSO control as described before. Quantification represents the integrated density of fluorescence per cell per field. Data represent pooled fields from 3 independent experiments (n>30 fields total). Mean ± SD. Unpaired t-test with Welch's correction. **(F)** Expression of the indicated CAF effector genes in HDFs treated with UT-155 versus DMSO, as in the previous panel, assessed by RT-qPCR. Data are expressed as fold change relative to DMSO. n=3 CAF strains. Mean ± SD. Two-tailed paired t-test. **(G)** Chromosome-specific TERRA expression in multiple CAF strains treated with Ostarine (10 μM, 48 h) versus DMSO. Results are expressed as fold changes relative to DMSO. n = 3 strains. Mean ± SD. Two-tailed paired t-test.**(H)** Quantitative RT–PCR analysis of TERRA transcripts originating from distinct subtelomeric regions (Chr1q, Chr7p, Chr9p, and ChrXYq) in two independent CAF strains (CAF1 and CAF7), corresponding to the same samples analyzed for AR protein levels in Fig. 2G. TERRA expression is shown as $2^{\wedge-\Delta Ct.}$ **(I)** RT-qPCR analysis of TERRA expression (Chr1q, Chr2q, Chr9p) in HDFs after AR silencing (shAR#57, shAR#18) versus control (plko) treated with ostarine (10 μM, 48 h) or DMSO as in the previous panel. Data are expressed as fold change relative to plko with DMSO. Mean ± SD. n = 3 biological replicates. One-way ANOVA with Šídák's multiple comparisons test.

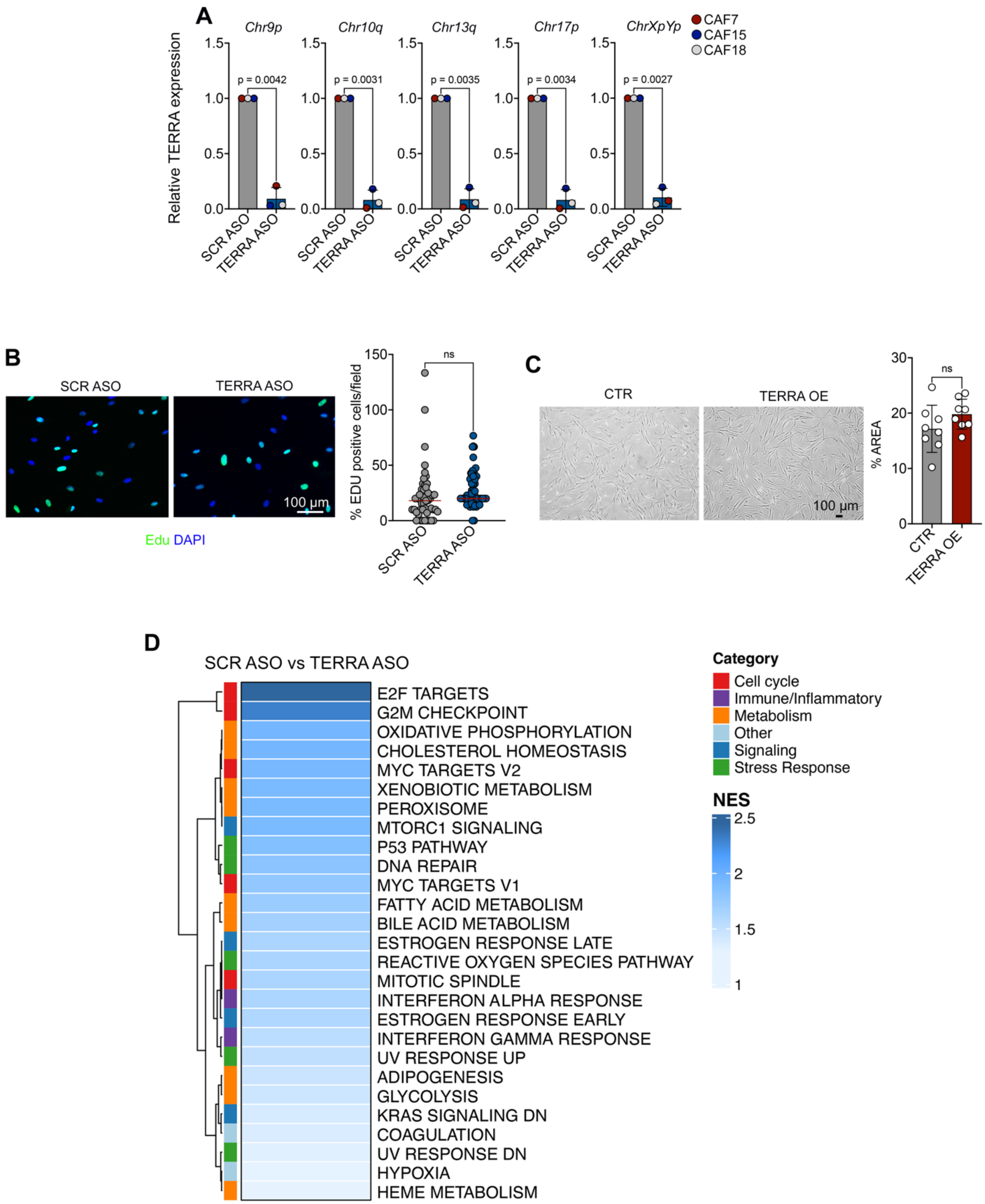

fig. S4. Effect of TERRA Silencing and Overexpression on CAF Proliferation and Transcriptional Program. (A) Chromosome-specific TERRA expression in CAFs 48 hours after

transfection with TERRA ASO or SCR ASO (100 nM). Data are expressed as relative fold change after GAPDH normalization. n=3 strains. Mean ± SD. Two-tailed paired t-test. **(B)** Proliferation of CAF transfected with TERRA ASO or SCR ASO assessed by EdU incorporation assay (green). Quantification shows the number of Edu-positive cells per field. Data represent pooled fields from 3 independent experiments (n>40 fields total). Mean ± SD. Unpaired t-test with Welch's correction. p=0.1194. **(C)** Bright-field images of the cell density of HDFs infected with TERRA OE lentiviral vector or CTR vector to assess the cellular proliferation. Quantification shows the percentage of area covered by the cells. Data represent pooled fields from 3 independent experiments (n=8 fields total). Mean ± SD. Unpaired t-test with Welch's correction. p=0.1645. **(D)** Gene Set Enrichment Analysis (GSEA) of the 50 Hallmark gene sets from MSigDB (https://www.gsea-msigdb.org/gsea/msigdb/), comparing CAFs treated with SCR ASO versus TERRA ASO. Shown are the gene sets significantly enriched in SCR ASO, ranked by Normalized Enrichment Score (NES). Functional categories are color-coded: cell cycle (red), immune/inflammatory (purple), metabolism (orange), signaling (blue), stress response (green), and other (gray). NES values are represented by the gradient scale on the right.

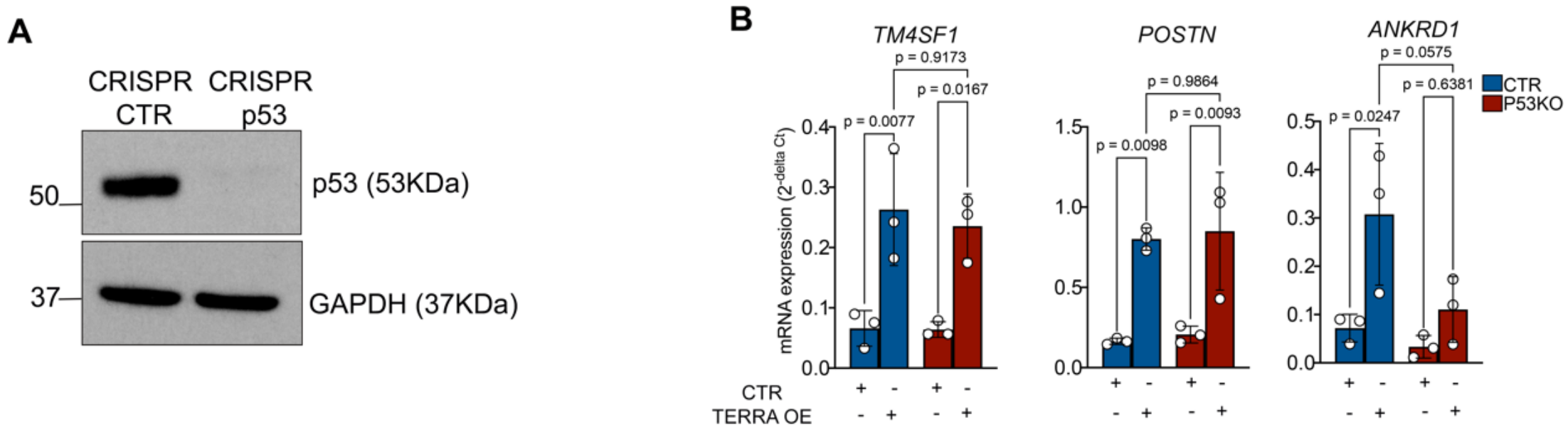

fig. S5 TERRA induces CAF gene expression independently of p53 status. **(A)** Western blot validation of p53 knockout HDFs18 generated using CRISPR–Cas9 with two independent sgRNAs targeting *TP53* (p53KO), compared to control CRISPR (CTR) cells. GAPDH serves as a loading control.
(B) Quantitative RT–PCR analysis of CAF effector genes (*TM4SF1, POSTN,* and *ANKRD1*) in control and p53KO HDFs following TERRA overexpression (TERRA OE). Gene expression is shown as $2^{\wedge-\Delta Ct}$. N=3 biological replicates. Mean ± SD. One-way ANOVA Šídák's multiple comparisons test

A

RNA binding	
NONO	*Non-POU domain-containing octamer-binding protein* (2.680)
TARDBP	*TAR DNA Binding Protein* (2.315)
PCBP3	*Poly(RC) Binding Protein 3* (3.945)
DHX9	*DEAH-Box Helicase 9* (2.463)
SON	*SON DNA-and RNA-binding protein* (1.797)
HNRNPCL1	*Heterogeneous nuclear ribonucleoprotein* (5.144)
HNRNPH2	*Heterogeneous nuclear ribonucleoprotein H2* (2.967)
HNRNPH1	*Heterogeneous nuclear ribonucleoprotein H* (2.106)
RPS15A	*ribosomal protein S15a* (14.873)
RPS3	*ribosomal protein S3* (7.264)
RPL7	*ribosomal protein L7* (1.232)
**Cytoskeletal**	
CLTC	*Clathrin Heavy Chain* (3.512)
FN1	*Fibronectin 1* (2.621)
PDLIM7	*PDZ and LIM Domain 7* (2.442)
CALD1	*Caldesmon 1* (1.326)

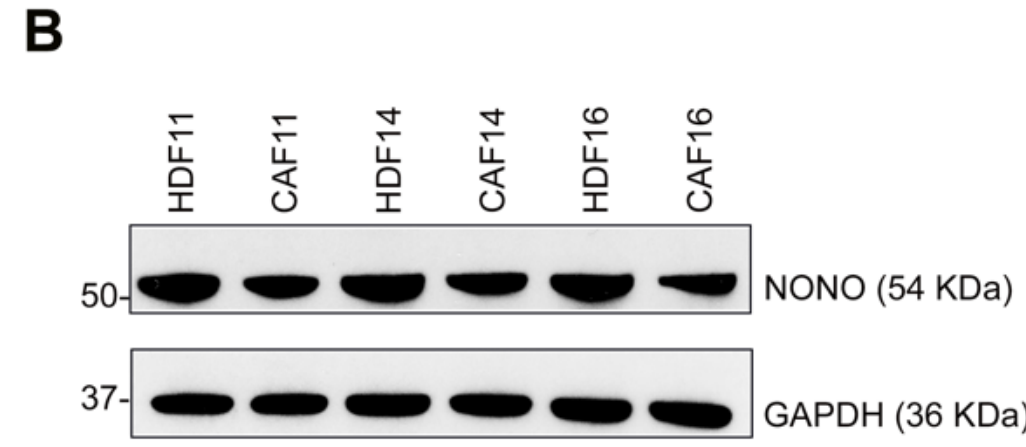

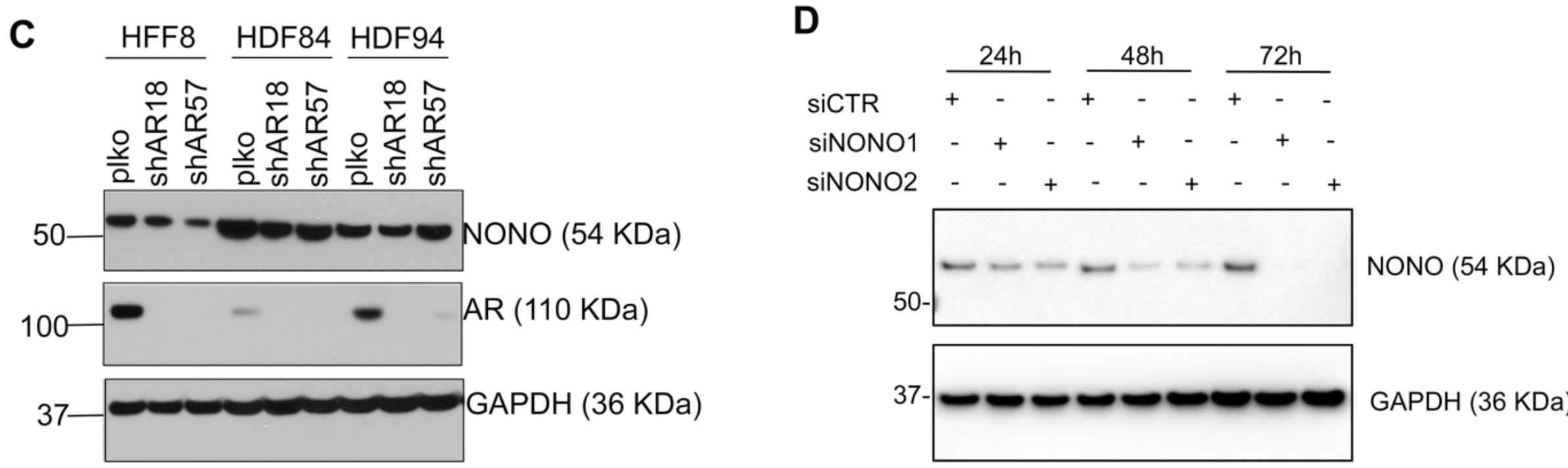

fig. S6 Expression and Regulation of NONO in CAFs and HDFs

(A) List of TERRA-interacting proteins commonly identified in two independent biological replicates of iDRIP-MS performed on CAFs (strain #19) following pulldown with a biotinylated TERRA antisense (TERRA-AS) oligonucleotide, compared to a negative control (TERRA-sense). Proteins were selected based on their detection in both replicates and filtered for Log_2 fold enrichment of TERRA-AS over TERRA-sense ≥ 1, calculated from the normalized intensity values of recovered proteins. Fold enrichment values are reported in brackets. **(B)** Immunoblot analysis with anti-NONO and GAPDH antibodies of CAFs compared to matched HDFs. n=3 strains. **(C)** Immunoblot analysis with anti-NONO, anti-AR, and GAPDH antibodies of HDFs after AR silencing (shAR#57, shAR#18) versus control (plko). n=3 strains. **(C)** Immunoblot analysis with anti-NONO and GAPDH antibodies of CAFs after NONO silencing (siNONO#1, siNONO#2) versus control (siCTR) 24, 48, and 72 hours after silencing.

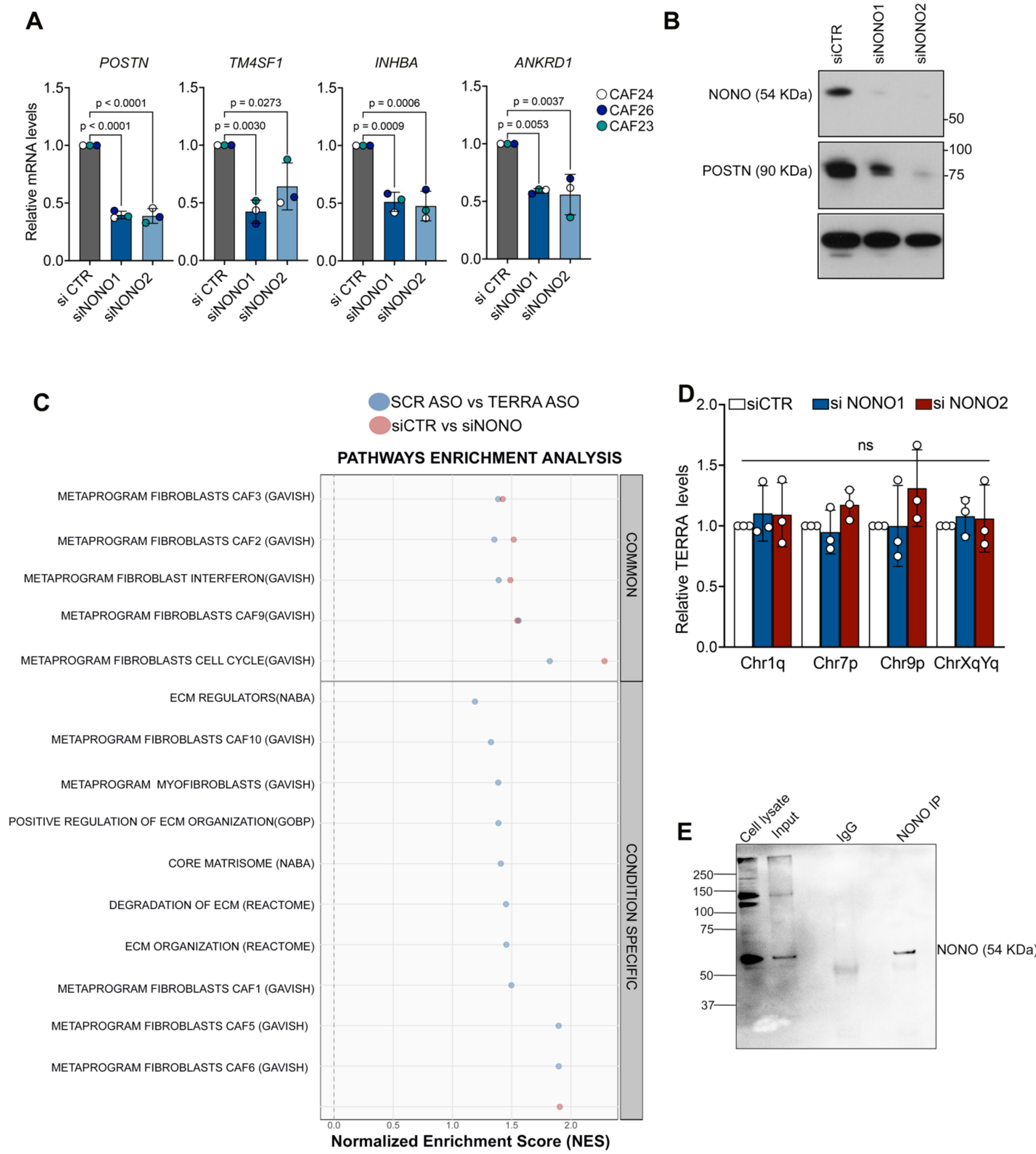
A
POSTN
TM4SF1
INHBA
ANKRD1
Relative mRNA levels
p < 0.0001
p = 0.0273
p = 0.0030
p = 0.0006
p = 0.0009
p = 0.0037
p = 0.0053
CAF24
CAF26
CAF23
si CTR
siNONO1
siNONO2
B
siCTR
siNONO1
siNONO2
NONO (54 KDa)
POSTN (90 KDa)
50
100
75
C
SCR ASO vs TERRA ASO
siCTR vs siNONO
PATHWAYS ENRICHMENT ANALYSIS
METAPROGRAM FIBROBLASTS CAF3 (GAVISH)
METAPROGRAM FIBROBLASTS CAF2 (GAVISH)
METAPROGRAM FIBROBLAST INTERFERON(GAVISH)
METAPROGRAM FIBROBLASTS CAF9(GAVISH)
METAPROGRAM FIBROBLASTS CELL CYCLE(GAVISH)
ECM REGULATORS(NABA)
METAPROGRAM FIBROBLASTS CAF10 (GAVISH)
METAPROGRAM MYOFIBROBLASTS (GAVISH)
POSITIVE REGULATION OF ECM ORGANIZATION(GOBP)
CORE MATRISOME (NABA)
DEGRADATION OF ECM (REACTOME)
ECM ORGANIZATION (REACTOME)
METAPROGRAM FIBROBLASTS CAF1 (GAVISH)
METAPROGRAM FIBROBLASTS CAF5 (GAVISH)
METAPROGRAM FIBROBLASTS CAF6 (GAVISH)
COMMON
CONDITION SPECIFIC
Normalized Enrichment Score (NES)
D
siCTR
si NONO1
si NONO2
ns
Relative TERRA levels
Chr1q
Chr7p
Chr9p
ChrXqYq
E
Cell lysate
Input
IgG
NONO IP
250
150
100
75
50
37
NONO (54 KDa)

fig. S7 NONO is required for TERRA-dependent CAF transcriptional program. (A) Expression of the indicated CAF effector genes (*POSTN, TM4SF1, INHBA, ANKRD1*) assessed by RT-qPCR in three CAF strains with or without NONO silencing, as described above. Data are expressed as fold change relative to siCTR and normalized to RPLP0. n = 3 strains. Mean ± SD. One-way ANOVA with Dunnett's multiple comparison correction. **(B)** Immunoblot analysis of CAFs (CAF19) 72 hours after transfection with control or two different NONO-targeting siRNAs. Blots were probed with antibodies against NONO, periostin (POSTN), and GAPDH (loading control).**(C)** GSEA was performed using curated gene sets from MSigDB (https://www.gsea-msigdb.org/gsea/msigdb/), including: i) extracellular matrix-related signatures (e.g., NABA matrisome (*66*), ii) fibroblast/CAF metaprograms (*67*), iii) Reactome degradation of ECM https://www.gseamsigdb.org/gsea/msigdb/human/geneset/REACTOME_DEGRADATION_OF_THE_EXTRACELLULAR_MATRIX, iv) GOBP_POSITIVE_REGULATION_OF_EXTRACELLULAR_MATRIX_ORGANIZATION (GO:1903055) Transcriptomic profiles were obtained from two CAF strains transfected with control or NONO-targeting siRNAs (siCTR vs. siNONO, red dots), and three CAF strains treated with control or TERRA-targeting antisense oligonucleotides (SCR ASO vs. TERRA ASO, blue dots). The x-axis indicates the Normalized Enrichment Score (NES). Pathways enriched in both conditions are grouped under "Common," while those specific to one condition are listed under "Condition Specific**."** **(D)** Chromosome-specific TERRA expression in CAFs 72 hours after transfection with two different NONO siRNAs (siNONO1; siNONO2) or siCTR (30 nM). Data are expressed as relative fold change after GAPDH normalization. n=3 biological replicates. Mean ± SD. One-way ANOVA Šídák's multiple comparisons test. **(E)** Representative Western blot showing NONO pulldown from CAF 24 used for ChIPmentation experiments. Cell lysate and 5% input fractions are shown alongside immunoprecipitation with anti-NONO antibody (NONO IP) and IgG control (IgG). A specific band corresponding to NONO (~54 kDa) is strongly enriched in the NONO IP compared to IgG, confirming efficient and specific NONO immunoprecipitation for downstream ChIPmentation analyses.

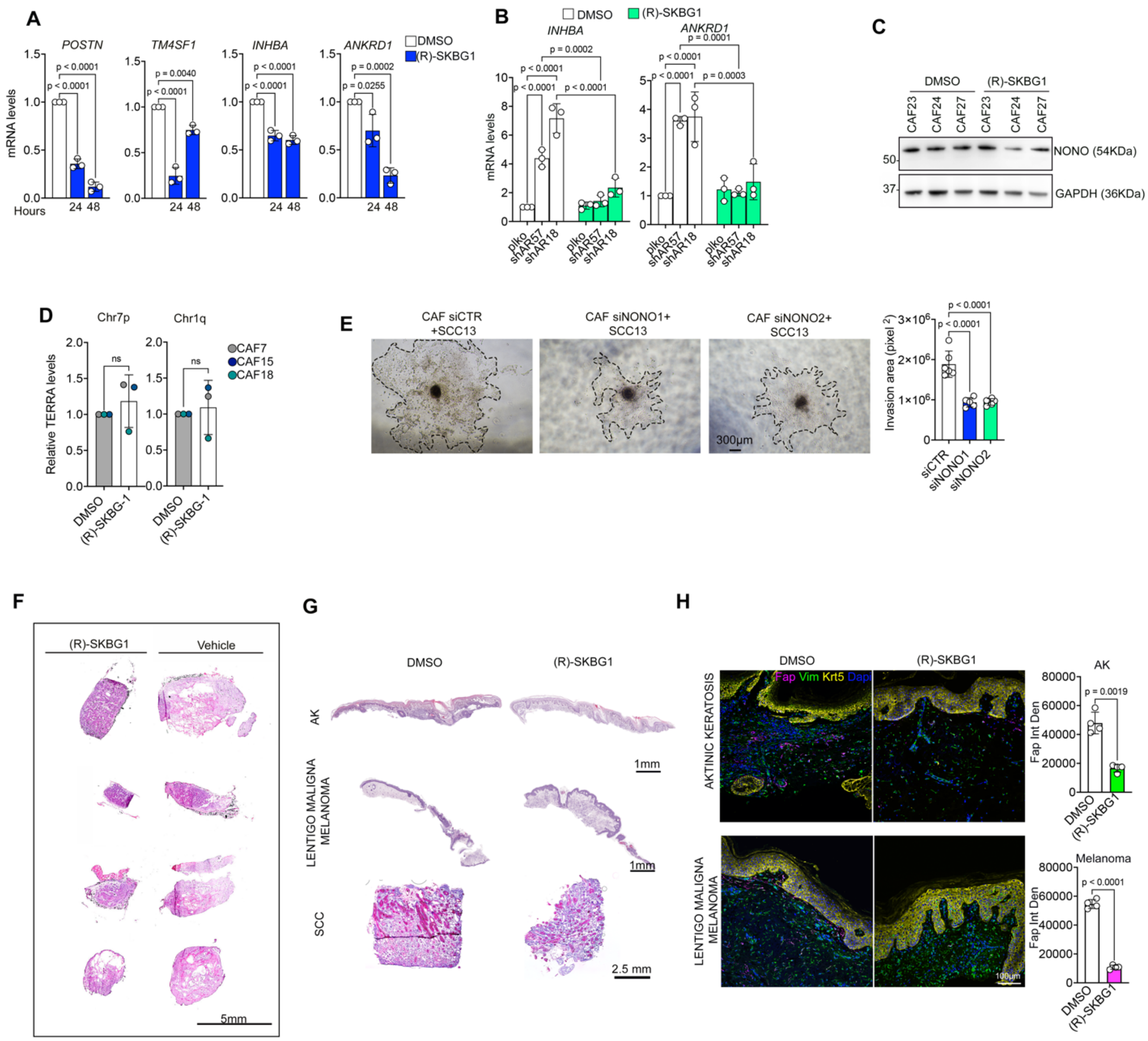

fig. S8 Pharmacologic and genetic disruption of NONO impairs CAF-mediated tumor progression. (A) Expression of the indicated CAF effector genes in CAFs with DMSO or (R)-SKBG1 treatment (5 µM) after 24, 48 hours, analyzed by RT-qPCR. Data expressed as fold change relative to DMSO. Data normalized to RPLP0.n=3 biological replicates. Mean ± SD. One-way ANOVA with Dunnett's multiple comparison test. **(B)** Expression of the indicated CAF effector genes in HDFs plus/minus AR silencing, followed by DMSO or (R)-SKBG1 treatment (5µM) for 48 hours, analyzed by RT-qPCR.Data expressed as fold change relative to DMSO. Data normalized

to RPLP0.n=3 biological replicates. Mean ± SD. One-way ANOVA with Šídák's multiple comparisons test. **(C)** Immunoblot analysis of anti-NONO and GAPDH antibodies of three strains of CAFs, 48 hours after DMSO or (R)-SKBG1 treatment (5 μM).**(D)** Chromosome-specific (Chr7p, Chr1q) TERRA expression in three CAF strains treated with DMSO or (R)-SKBG1 as described before. Data expressed as fold change relative to DMSO, normalized to GAPDH. Mean ± SD. Paired two-tailed t-test; p-value=0.476 (Cht7p), p=0.7145 (Chr1q).**(E)** Spheroid invasion assays of SCC13 cells admixed with CAFs transfected with siCTR, or siNONO (1,2). Shown are representative bright field images of spheroids and quantification of the invasion area measured as the difference between the core and the surrounding invaded area delimited with dotted lines. n >5 spheroids per condition. One-way ANOVA with Dunnett's multiple comparisons test. **(F)** H&E-stained tumor (#2, #3, #4, #5) as described in Fig.7F.(**G**) H&E staining of the ex vivo actinic keratosis, cSCC, and lentigo malignant melanoma treated with DMSO or (R)-SKBG1 (10μM) for 48 hours. **(H)** Representative immunofluorescence images of actinic keratosis (AK) and lentigo maligna melanoma biopsies treated ex vivo with DMSO or the NONO inhibitor (R)-SKBG1 (10 μM) for 48 h. Sections were stained for FAP (Fap; magenta), vimentin (Vim; green), keratin 5 (Krt5; yellow), and DAPI (blue). Lower panels: quantification of FAP immunofluorescence signal intensity (integrated density) in AK and melanoma lesions. Each point represents one field. Mean ± SD; Unpaired t test with Welch's correction.

Table S1. List of Antibodies

**Anti-Vimentin**	Rabbit	Abcam ab45939
**Anti-Vimentin**	Rat	R&D MAB2106
**Anti-Cytokeratin Pan**	Mouse	Invitrogen MA5-13203
**Anti-POSTN**	Mouse	Santa Cruz sc-398631
**Anti-Fap**	Rabbit	Cell Signalling 66562T
**Anti-NONO**	Rabbit	Proteintech 11058-1-AP
**Anti-NONO**	Mouse	Sigma
**Anti-AR**	Rabbit	Millipore CS207339
**Anti-p53**	Mouse	Abcam ab1101
**Anti-AR**	Mouse	Santa Cruz CS207339

Table S2. List of primers

**RT-qPCR primers**	**Primers (5′→3′)**
TERRA RT-primer	CCCTAACCCTAACCCTAACCCTAACCCTAA
GAPDH RT-primer	GCCCAATACGACCAAATCC
1q TERRA	Forward: GCATTCCTAATGCACACATGAC;<br>Reverse: ACCCTAACCCGAACCCTA
2q TERRA	Forward: AAAGCGGGAAACGAAAAGC;<br>Reverse: GCCTTGCCTTGGGAGAATCT
7p TERRA 2	Forward: GAGAGAGGGTTTCACTCTGTTG;<br>Reverse: GGTGGTTCACGCCTGTAAT
9p TERRA	Forward: GAGATTCTCCCAAGGCAAGG
10q TERRA 2	Forward: ATGCACACATGACACCCTAAA;<br>Reverse: TACCCGAACCTGAACCCTAA
13q TERRA 2	Forward: CTGCCTGCCTTTGGGATAA;<br>Reverse: AAACCGTTCTAACTGGTCTCTG
17p TERRA 1	Forward: CTTATCCACTTCTGTCCCAAGG;<br>Reverse: CCCAAAGTACACAAAGCAATCC
XpYp TERRA 3	Forward: AAGAACGAAGCTTCCACAGTAT;<br>Reverse: GGTGGGAGCAGATTAGAGAATAAA
GAPDH	Forward: AGCCACATCGCTCAGACAC;<br>Reverse: GCCCAATACGACCAAATCC
RPLP0	Forward: GCAATGTTGCCAGTGTCTGT;<br>Reverse: GCCTTGACCTTTTCAGCAAG
INHBA	Forward: ATTTCCTGTGCACCTTTTGG;<br>Reverse: CAAACCTTCTGCCCACATCT
AR	Forward: TTGTGTCAAAAGCGAAATGG;

	Reverse: AGTCAATGGGCAAAACATGG
NONO	Forward: CATCAAGGAGGCTCGTGAGAAG;<br>Reverse: TGGTTGTGCAGCTCTTCCATCC
MMP1	Forward: ACACCTCTGACATTCACCAAG;<br>Reverse: ATGAGCCGCAACACGATG
POSTN	Forward: AAGGGAGTAAGCAAGGGAGA;<br>Reverse: GCATTTTTGTCCCGTATCAG
TM4SF1	Forward: TTTCTGGCATCGTAGGAGGT;<br>Reverse: CCAGCCCAATGAAGACAAAT

Chipmentation Primers	Site	FORWARD PRIMER	REVERSE PRIMER
**Chr1q**	S1	GCAGCATTCTTCTCAGCACA	AGGGCAGCAACCGGGAAA
	S2	CCACCACTATACTGCGAGCA	ACTGGCGACTAGGACAACTG
	S3	GGTGTGCTGCCACTACAATG	GGCAGGGCTCTCTTGCTC
	S4	TTTTCAATGAAGTTATCTTCCATCTTT	AAAGGAGCTATTACTGCGGAGA
	S5	TGCCCCAATCATCATTCACTC	ACTTATGGTGCCAGGTTTCTC
	S6	TTTTCACAACTTCCTTTGTTAGC	GCCACCCCAAAATATAATGGTA
	S7	GGTGGAGTTTTCAGCCCTCT	GCCTGATAAGAGACCACCCA
	S8	CACCCACCCAGTGAGAGATT	CCTCCTCCTTGAAGCATCTG
**Chr7p**	S1	CCAAAGTACACAAAGCAATCCG	TGAAATTGGGATGTGATCAGGA
	S2	GGGAGTCCAGACAGAAAACA	TCCCTTTATGTGGATAGCAAGTT
	S3	ACTCTCAACACTCACAGAACAC	GATTGCACTGTAGGACACGC
	S4	GAATGGCATGCAGGGGATG	TCAATTTACAACCTGGCTCTGC
	S5	TCTTGTGTTGGGAGAGCAAC	AGAGGTGCTGATGGTTGAGT
	S6	ATTCTCTCATGACAAGGTTGCT	TGTGTCTTTCGTACGCTAATGA
	S7	TCATTAGCGTACGAAAGACACA	TCCGTTTTATAGGTTGTTGGAAC
	S8	ACAACGCTGATTTCCTGATCT	AGTACAGCTTGTTTCTTCCCTT
	S9	TTTCTTCCTCTGCTGAGTGCT	GGGGTCGTGTTAATTTGGTG
	S10	GGTTCAGCACAGACGCTCTT	CCAAAATTATGACACTGAATAAACTGA

IL6 Site 1	TTCCAAAGATGTAGCCGCCC	CCGTCGAGGATGTACCGAAT
IL6 Site 2	AGTGAGGAACAAGCCAGAGC	TGGCATTGCATCCCTGAGTT
TM4SF1 Site 1	CACAGATGGCTGAAAATAAACAGCAGAGAATTTGGATTCAAAACTGTTGA	TTTAGGCAGAGAACCAGCCAACCCCTTCCGGCTCCCCAGAGCAAATGTTA

TM4SF1 Site 2	GATGCATCGTGCACACTTCC	TGTTTCTCACATCCTGCCCC
POSTN site 1	CTTGGTCCCGACCCCTGATA	TTCGGGGACTAACTGCAACG

Data File S1: TERRA-interacting proteins identified by iDRIP-MS in CAFs across two independent experiments.
